# Supplementary material for: Isoform‐resolved correlation analysis between mRNA abundance regulation and protein level degradation
Source: Mol Syst Biol. 2020 Mar 16;16(3):e9170. doi: 10.15252/msb.20199170 (PMC7073818; doi:10.15252/msb.20199170)
Supplement: Supplementary file 1 — Appendix [file MSB-16-e9170-s001.pdf]

Contents

Supplementary Note 1: Step-by-step protocol for pSILAC data analysis in Spectronaut. The document provides a recommended protocol for pulsed SILAC data analysis. (Page 2-6)

Supplementary Note 2: Optimized pulsed SILAC DIA-MS data analysis. The document describes optimized spectral library generation, targeted data analysis workflow selection, and a new function enabling the usage of the b-ions in labeled DIA-MS data analysis using Spectronaut. (Page 7-11)

Supplementary Note 3: Comparison between the **pSILAC-MS1**, **pSILAC-TMT**, and **pSILAC-DIA** approaches for protein turnover analysis. (Page 12-15)

Supplementary Note 4: Comparison between the relative isotope abundance (RIA)-based and normalized light intensities (NLI)-based k_{loss} calculations (Page 16-19)

Supplementary Methods (Page 20-22)

Supplementary Figures captions (Page 23-25)

Supplementary References (Page 26-27)

Supplementary Figures S1-S8 (Page 28-35)

Supplementary Note 1: **Step-by-step protocol for pSILAC data analysis in Spectronaut**

A. Library Generation

1) Select files for library generation. Both DDA and DIA data are supported. For optimal results, consider adding SILAC labeled mass spectrometric measurements.

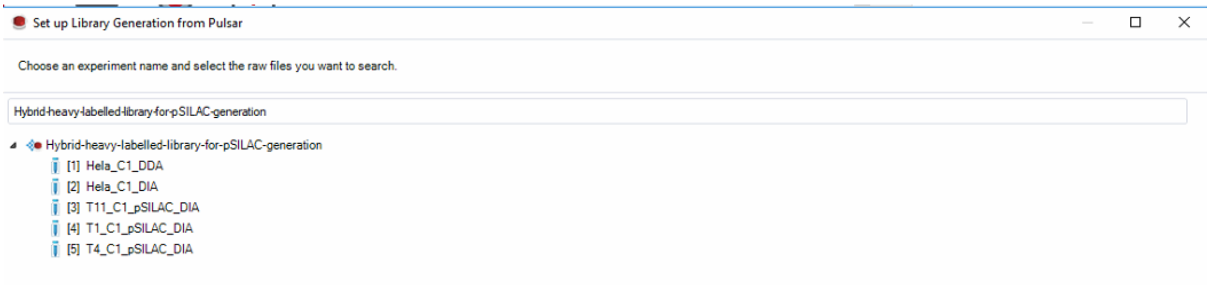

2) Select a FASTA database of protein sequences.

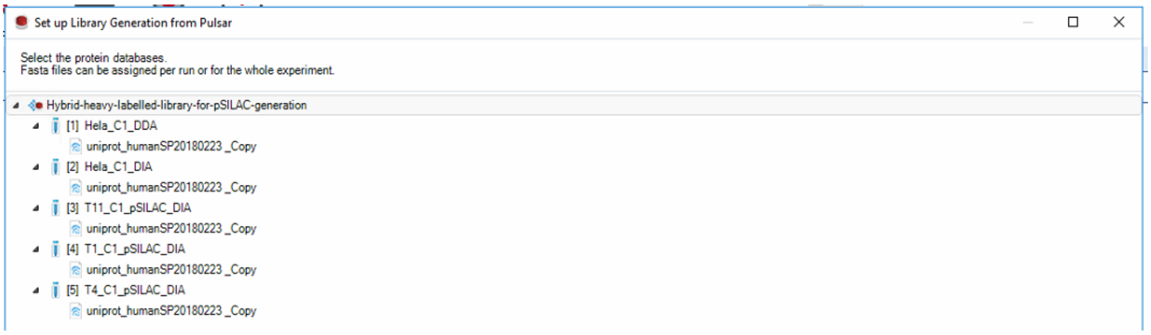

3) Select search settings. For label-free data, BGN Factory settings can be used. For pSILAC data, labels must be specified in the "Labeling" settings.

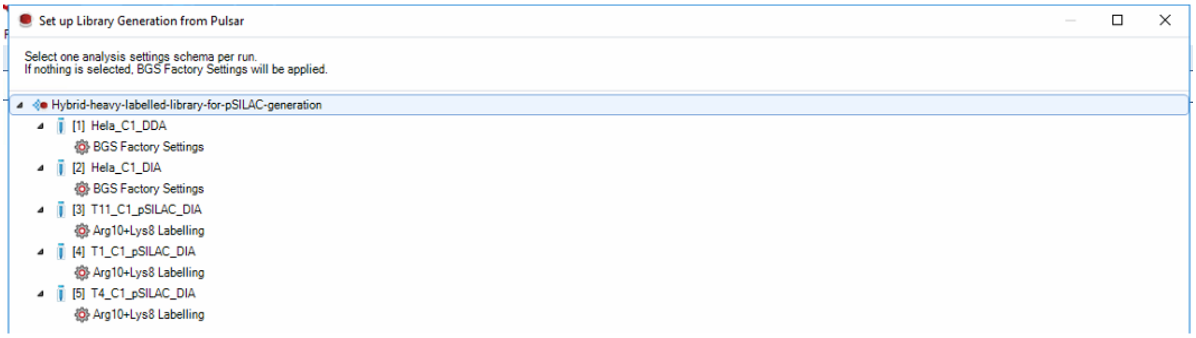

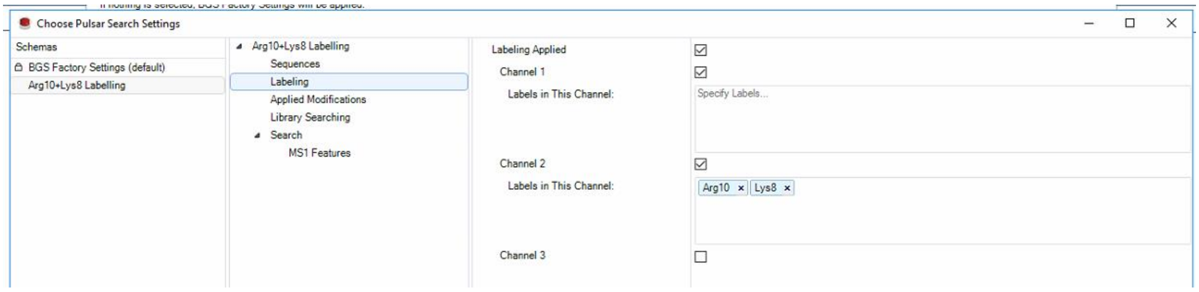

4) Optionally, previous searches can be added from Search Archives to further increase the library size, with the identification FDR globally controlled at the end.

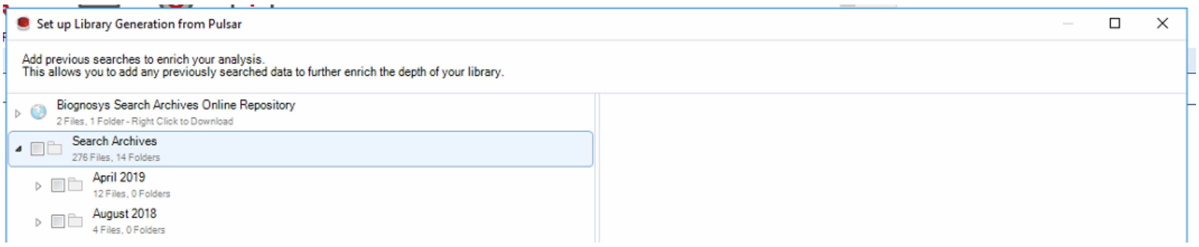

5) Choose the settings for library generation. To ensure the complete labeling of the final assay library, the "**In-Silico Generate Missing Channels**" option must be enabled in the Workflow settings. The workflow needs to be specified as "label".

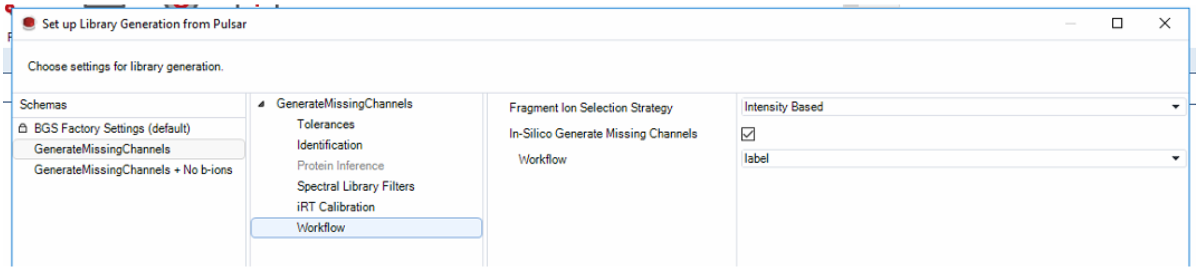

6) Check the analysis summary and run the library generation.

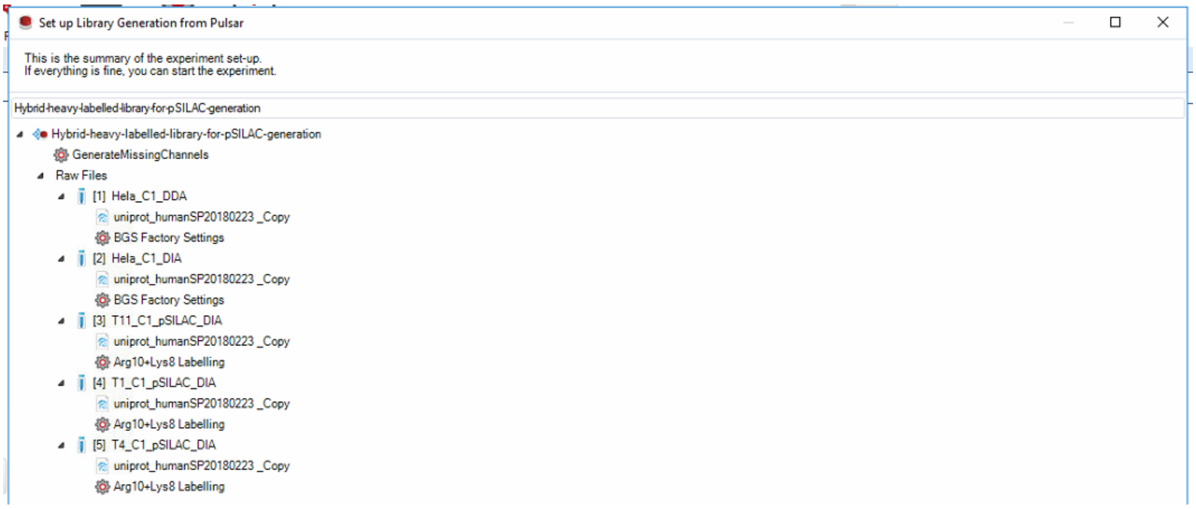

B. Targeted data search and extraction

1) Select DIA files to analyze and the previously created library.

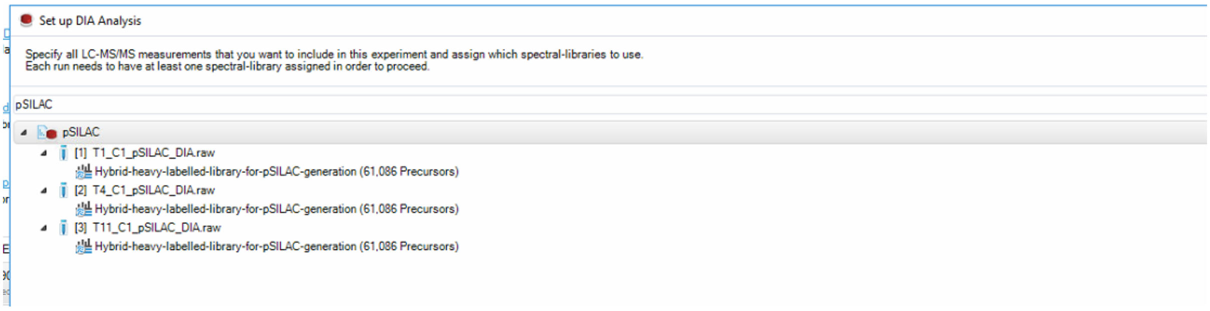

2) Select the search and extraction settings for DIA analysis. The "**Inverted Spike-In workflow**" (ISW) is recommended for the pSILAC data analysis. To do so, in the Workflow settings, the "Spike-In" workflow is selected in Multi-Channel Workflow Definition. Both "Inverted" and "Reference based Identification" options are enabled. In the quantification settings, the "Interference correction" option is enabled with minimal 3 fragment ions to keep. The "**Exclude All Multi-Channel Interferences**" option has to be enabled to exclude ions that might interfere and cause ratio distortion in the labeled workflows. For example, this option enables the usage of b-ions falling in different isolation windows for pSILAC data quantification (as described in the **Supplementary Note 2**).

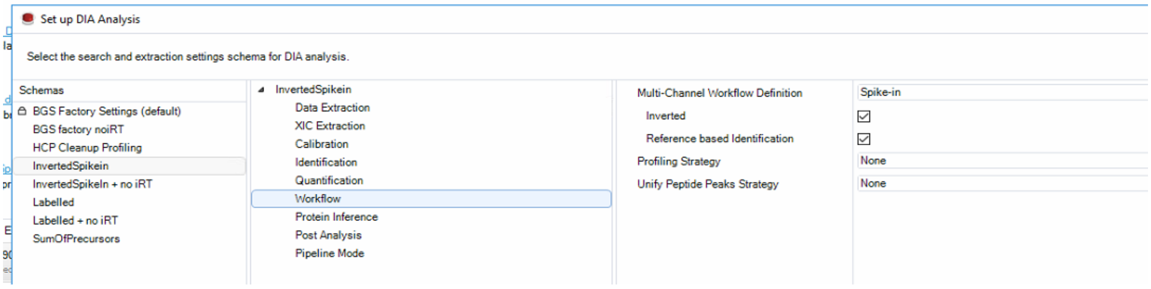

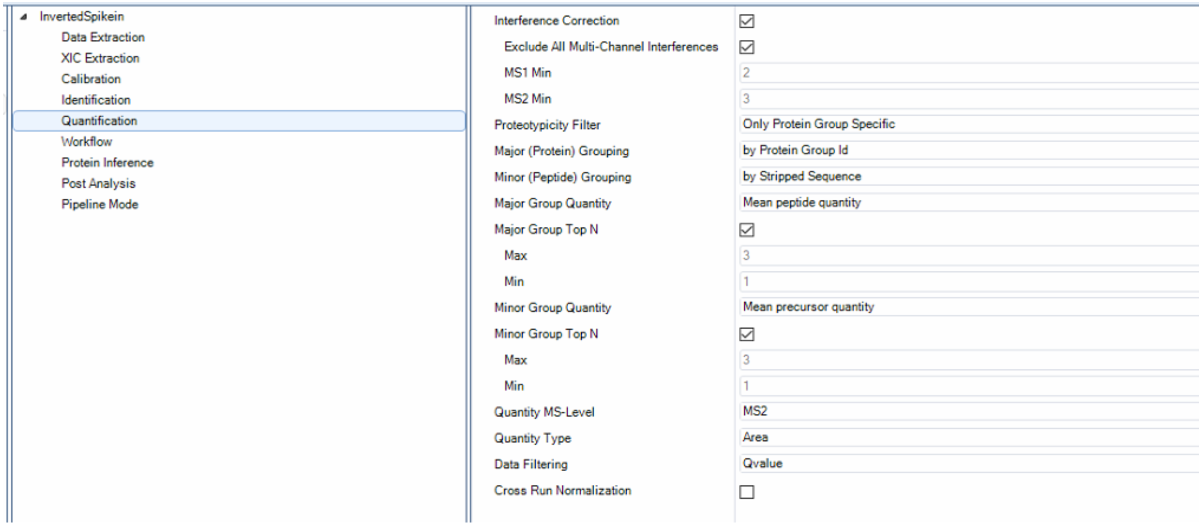

3) Select the same FASTA database of protein sequences, check, and run the analysis.

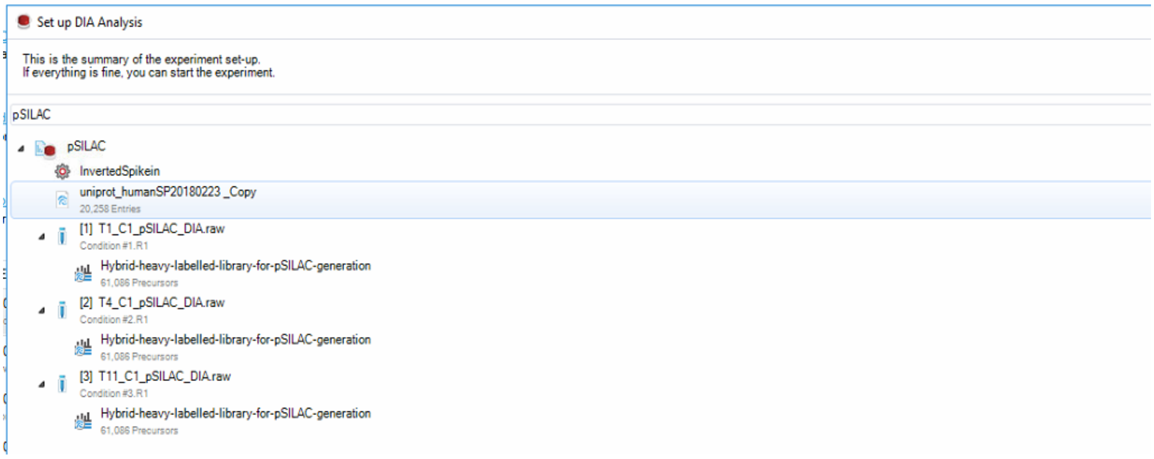

4) The „heavy" (target) and „light" (reference) fragment ion signals can be conveniently visualized in the Analysis perspective using the "XIC graphic" plot, which has been also optimized for labeled workflows. In this plot, the upper part corresponds to the „light" signal (reference) while the lower part shows the signal of the „heavy" (target) channel in retention time and within the peak boundaries identically defined for both channels. Interfering ions discarded by the quantification filtering are marked with dashed lines. The XIC graph can show either MS1 or MS2 quantification based on user preference (in the right-click menu). An example of a peptide quantified in three time points is given below. The intensity of the target channel (depicted below the zero line) is increasing over time while the „light" ion intensity is decreasing.

MS2 XIC

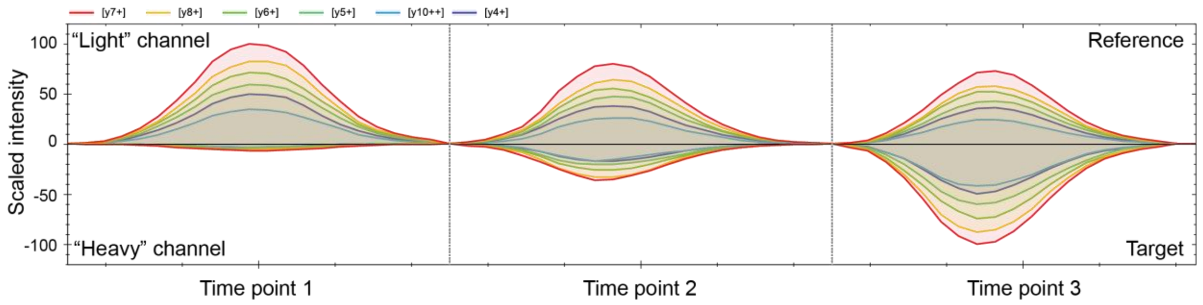

MS1 XIC

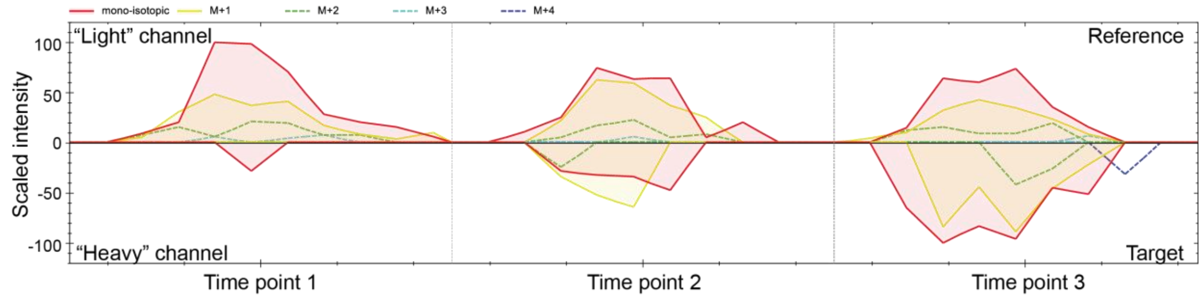

5) For the downstream analysis, the intensities of the „heavy" (target) and the „light" (reference) peptide precursors as well as the H/L ratio ("target/reference ratio") after the quantification data filtering can be conveniently reported by the pivot report in the Report perspective.

EG.PrecursorId – unique peptide precursor id

EG.ReferenceQuantity(Settings) – „light" signal intensity

EG.TargetQuantity(Settings) – „heavy" signal intensity

EG.TargetReferenceRatio(Settings) – the heavy/light ratio

These values can be further used as the input values for turnover estimation and following bioinformatics analyses.

Supplementary Note 2: Optimized pulsed SILAC DIA-MS data analysis

A. Library selection for pulsed SILAC data analysis

This Note further supports the **Supplementary Note 1**. Spectronaut currently can combine label-free data and isotopically-labeled data, either acquired by using DDA-MS or DIA-MS, to generate a hybrid sample-specific and heavy-labeled library in one step with proper global FDR control. Thus, the coverage of the library can be increased by using comprehensive sample-specific assays. First, data are searched using Pulsar and "directDIA" with appropriate settings for the label-free and labeled data. Then, a complete labeling of the hybrid library is achieved through the "**In-Silico Generate Missing Channels**" option, which detects the labeling setup of an experiment and applies this labeling to the whole assay library to ensure each one of the peptides is represented by both „light“ and „heavy“ transitions. Another option is to generate a library using label-free DIA and DDA data only and label the library *in-silico* using the "**Generate Labeled Library**" function in the library perspective of Spectronaut.

To test whether the hybrid library performs better than the label-free-only library in our pSILAC data, we performed a systematic evaluation of four different libraries (**Table 1)**. Libraries 1 and 2 (L1 and L2) were created using label-free DIA and DDA data only. On the other hand, for the libraries 3 and 4 (L3 and L4) we also included pSILAC data raw files to create a "hybrid" library. Furthermore, in L2 and L4 only y-ions were used while all the b-ions were removed. Thus, the libraries differed in two main parameters – whether the labeled data were included and whether the b-ions were kept or removed. For the purpose of the library and workflow testing, fifteen raw files were included. HeLa cell lines 1, 2, 3, 4, and 5 were used in all three time points of a pSILAC experiment (1, 4.5, and 11 hours, or T1, T4, and T11).

Table 1: *Overview of libraries tested for pSILAC data analysis*

lib #	# of raw files used	label-free DDA+DIA data used	pSILAC data used for the library	b-ions included
**Library 1**	122	YES	NO	YES
**Library 2**	122	YES	NO	NO
**Library 3**	150	YES	YES	YES
**Library 4**	150	YES	YES	NO

To evaluate the results, we first compared the size of the four libraries. As shown in **Figure 1 (hereafter, the "Figures" are referred to those in this Note, not the main Figure nor Supplementary Figures)**, there was a minor difference (1% – 2% of ids) between the libraries.

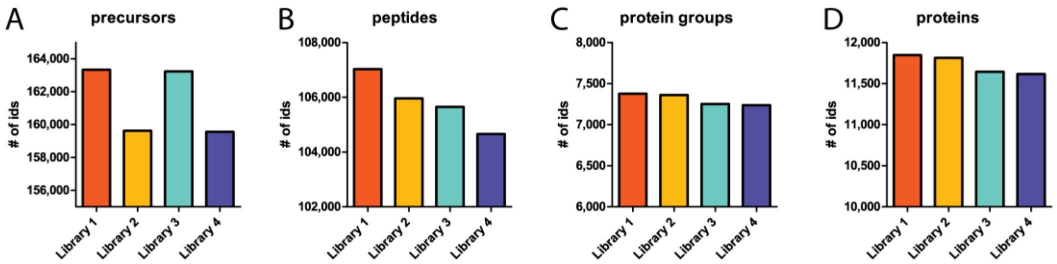

***Figure 1:** Size of the libraries created using **label-free data only** (Library 1 and 2) and **label-free data combined with pulsed SILAC data** (Library 3 and 4). The numbers of peptide precursors **(A)**, unique peptide sequences **(B)**, protein groups **(C)**, and proteins **(D)** are shown.*

Secondly, two libraries were used for targeted pSILAC data analysis, L2 and L4, to evaluate which library – *in-silico* labeled or hybrid - provides the best identification and quantification for the downstream analysis. For data extraction, the Inverted Spike-In workflow (ISW) was used. The number of valid target/reference ratios (or H/L ratios) reported was higher in all 15 raw files subjected to the analysis independently on the cell line or time point (**Figure 2A**). Consequently, the number of calculated peptide and protein k_{loss} values was larger: the number of peptide k_{loss} values increased by 6.2% whereas the number of protein k_{loss} increased by 3.9% (both reported values are average from the five cell lines) (**Figure 2B** and **2C**). Thus, the sample-specific hybrid library provided better quantification results and was used for the data analysis presented in the main text.

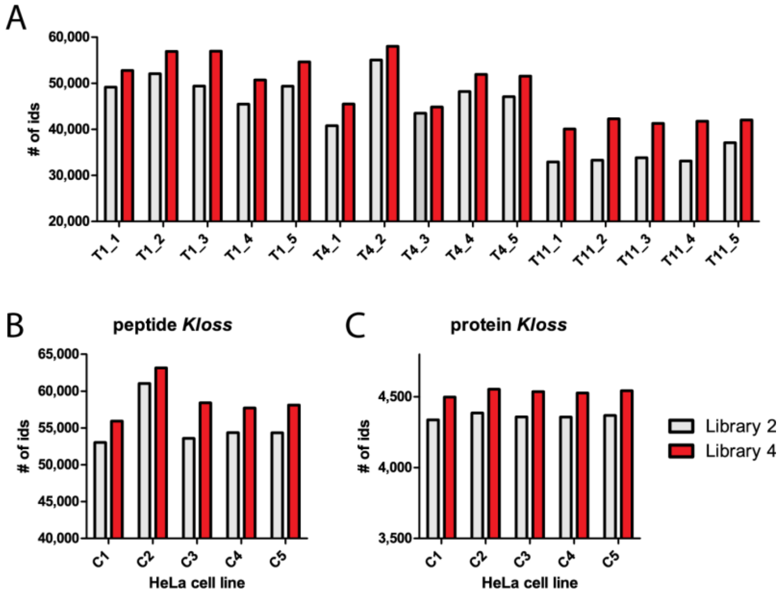

***Figure 2: Sample-specific hybrid library provided better quantification results than the in-silico labeled library**. Quantitative comparison of the data extracted using in-silico labeled library (Library 2) and a hybrid library (Library 4). **(A)** The number of valid target/reference (H/L) ratios reported by Spectronaut for each one of the raw files; T1, T4, and T11 indicate time points 1, 4.5, and 11 hours. Peptide **(B)** and protein **(C)** k_{loss} values were calculated as described in the Methods section of the main text; only ids with increasing „heavy" isotopic signals over the time points and ids quantified in at least two time points were included. Number if ids in the five HeLa cell lines are shown.*

B. Selection of the workflow for pulsed SILAC data analysis

We also tested the usage of the Inverted Spike-In workflow (ISW) and the “Labeled” workflow (LBL) for pSILAC data analysis. Notably, the peak picking and identification in the LBL is performed by using both „light“ and „heavy“ precursors while ISW (with "Reference based Identification" option selected) does both the peak-picking and identification based on the „light“ precursors. Moreover, all labeled workflows assemble the heavy-versus-light elution by using the same peak boundaries before DIA identification, so that no post feature alignment (Rost et al, 2016) is required for labeling DIA analysis.

Due to the peak picking algorithm, ISW can improve the reliability of detection of newly synthesized „heavy“ protein in the early time points during labeling, when the „heavy“ signals are dramatically lower than the „light“ ones from those pre-existing proteins. To test whether this feature can improve pSILAC data analysis, we further evaluated the outcome of LBL and ISW workflows in the five HeLa cell lines (**Figure 3**). The hybrid Library 4 was used for this comparison. As expected, the ISW significantly outperformed the LBL workflow in the two earlier time points (**Figure 3A**). Overall, ISW also provided k_{loss} values for 6.2% more peptides and 6.5% more proteins (**Figure 3B** and **3D**). We thus used ISW for pSILAC data analysis.

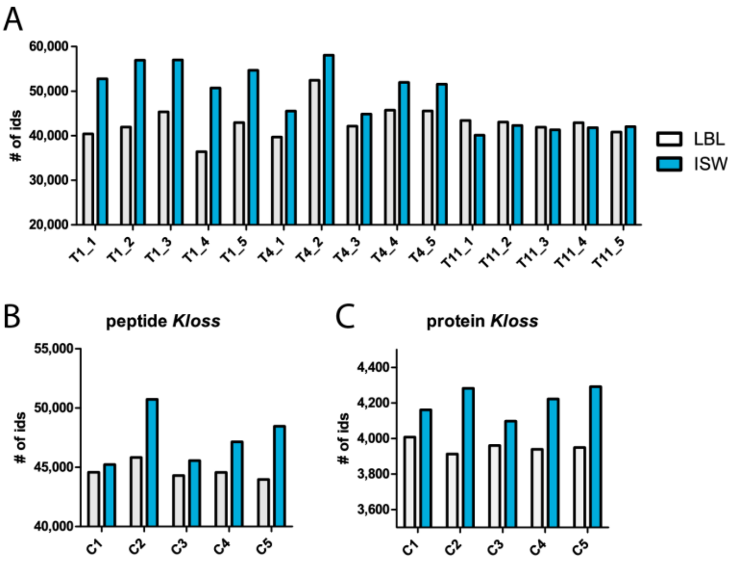

Figure 3: The Inverted Spike-In workflow provided better quantification results than the labeled workflow. *Quantitative comparison of the data extracted using the Labeled (LBL) or Inverted Spike-In workflow (ISW). Library 4 was used for the analysis.* ***(A)*** *The number of valid target/reference (H/L) ratios reported by Spectronaut for each one of the raw files; T1, T4, and T11 indicate time points 1, 4.5, and 11 hours. Peptide* ***(B)*** *and protein* ***(C)*** k_{loss} *values were calculated as described in the Methods section of the main text; only ids with increasing „heavy“ isotopic signals over the time points and ids quantified in at least two time points were included. Number if ids in the five HeLa cell lines are shown.*

C. Improving the identification and quantification by including the b-ions for the pSILAC data analysis

In our previous work, we strictly excluded the b-ions during pSILAC or SILAC data analysis to avoid interferences and corresponding ratio distortion caused by b-ions not carrying the „heavy“ labels in case the „light“ and „heavy“ peptide precursors are fragmented in the same isolation window (Liu et al, 2017; Liu et al, 2019). However, in case the b-type fragment ion either contains a heavy-label (e.g., due to a missed cleavage) or the „light“ and „heavy“ precursors fall in a different isolation window, these ions theoretically could be still used to improve identification and quantification.

To enable the usage of all fragment ion types in the isotopically-labeled data analysis, a new "Exclude All Multi-Channel Interferences" option is now available in Spectronaut v13. This function automatically recognizes ions that show channel interference in the labeled workflows, and these are completely removed from quantification. The function was evaluated in a smaller test (only HeLa 1 data were included). By enabling the b-ions, the number of peptide and protein ids increased by 14% and 11.2%, while the quality of the data (i.e., H/L ratio distribution in different time points) seems to be not affected when compared to the results of the analysis completely excluding b-ions (**Figure 4**).

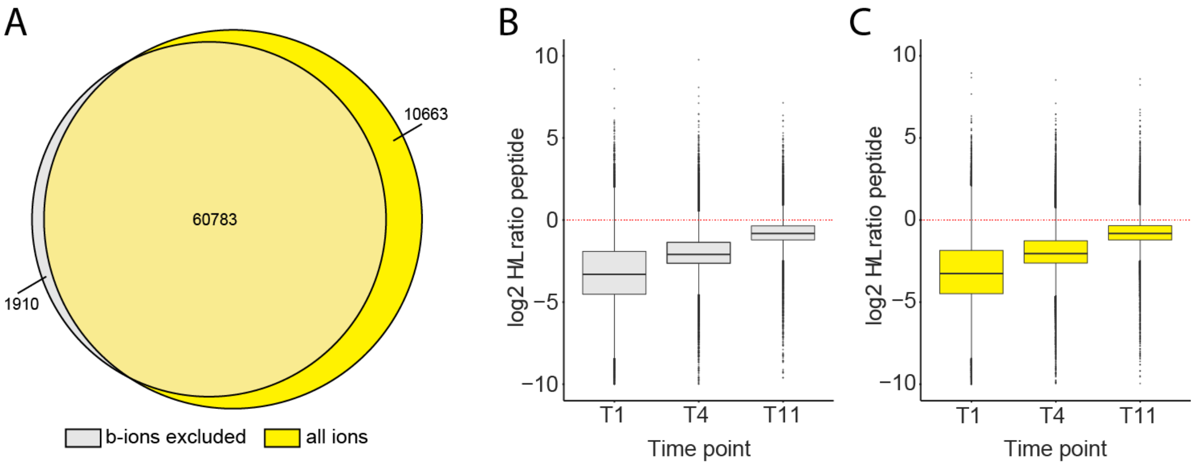

Figure 4: The usage of b-ions together with the "Exclude All Multi-Channel Interferences" option improves pSILAC data identification and quantification**.** ***(A)*** *The Venn-diagram shows the number of peptide ids shared between and exclusive for two data sets generated either with b-ions completely removed from the library and analysis ("****b-ions excluded****") or with all ions included in the library and analysis ("****all ions****"). Log*$_2$ *H/L ratio distribution of peptides across three time points in the "b-ions excluded"* ***(B)*** *and "****all ions****"* ***(C)*** *data sets.*

D. Summary

- The sample-specific hybrid library provided better identification and quantification than the *in-silico* labeled library generated from label-free data exclusively.
- The “Inverted Spike-In” workflow outperformed the “Labeled” workflow, especially in the early time points of the pulse-chase SILAC labeling.
- A new option is now available in Spectronautv13, called “Exclude All Multi-Channel Interferences”, so that the users can benefit from using the b-ions in their analysis.

Supplementary Note 3: **Comparison of pSILAC-DIA to pSILAC-MS1 and pSILAC-TMT approaches for protein turnover analysis.**

Below we compared our pSILAC-DIA data to two alternative methods for the proteome-wide turnover measurement by pSILAC experiment – namely pSILAC-MS1 and pSILAC-TMT. Both methods are often used. For example, Savitski and colleagues are the major developers of the methods and software for the multiplexed TMT-based workflow analyzing protein thermal stability (Franken et al, 2015) and dynamics. In 2018, they published a systematic analysis on protein turnover in primary cells using an optimized pSILAC-MS1 workflow (Mathieson et al, 2018) and a study uncovering proteolysis targeting chimeras (PROTACs) effects using a pSILAC-TMT workflow (Savitski et al, 2018).

<u>pSILAC-DIA vs. pSILAC-MS1</u>

In pSILAC-MS1 approach, the same pSILAC experiment is performed, whereas the SILAC heavy/light ratios are inferred from MS1 isotopic pairs following a shotgun proteomic workflow.

1) In Mathieson et al., with the extensive, gel- or high-pH-based fractionations coupled mass spectrometry quantification, 4000-6000 proteins were on average identified in each of the several primary cell types (Mathieson et al, 2018) (a further check on their supplementary table suggests ~2200 proteins were quantified with turnover estimates across cell types). This number is very close to our DIA data reported here without any fractionation before MS analysis. We suggest that the nature of DIA (or SWATH-MS in our previous papers (Liu et al, 2017; Liu et al, 2019)) generates much less missing values than traditional shotgun (or DDA) measurement, and thus favorably supports those experiments involving multiple (or large number of) samples to be measured, such as the ones with multiple time points following pSILAC design.
2) In Mathieson et al, the authors had to optimize the data analysis workflow of pSILAC-MS1 by two innovations (Mathieson et al, 2018). **a)** They improved the theoretical MS1 isotope-fitting algorithm, because the MS1 ions features have multiple, (possibly) high charge-states. **b)** They improved the MS1 isotope dissection to ensure the quantification quality (especially for early time points), particularly because certain co-eluting interfering isotopic clusters are indistinguishable. Although these improvements were impressive as compared to the traditional pSILAC-MS1 experiments, they are currently only available with isobarQuant search engine and package (Mathieson et al, 2018).

In our pSILAC-DIA, correspondingly to above, **a)** With the new **Appendix Figure S2**, we have shown that MS2 based DIA quantification has much less and more uniformly distributed charge states (83.08% are charge 1, and the other 15.92% are charge 2, **Figure S2A***)* than the MS1 results (**Figure S2B**), which greatly reduced the isotope-fitting difficulty for calculating theoretical isotopic envelope. **b)** Furthermore, because all high-resolution DIA-MS2 peak groups (with 30k in Orbitrap) are aligned and identified at MS2 level, the „heavy“ and „light“ signals are well matched. Importantly the DIA isolation window schema and the MS2 level acquisition greatly reduced the noise background from a full mass-

range MS1 level acquisition. These factors together ensure that the pSILAC-DIA data has a significantly better quantitative accuracy and reproducibility than the direct MS1-based quantification (even at 120k) (as compellingly shown in Appendix **Figures S3** and **S4**), especially for early pulse-chase time points which are known to heavily impact the protein turnover calculation (Claydon & Beynon, 2012).

In summary, the pSILAC-DIA quantification successfully improved the quantitative accuracy and reduced the MS data procession difficulty in pSILAC experiment, as compared to pSILAC-MS1 approach.

pSILAC-DIA vs. pSILAC-MS1 and pSILAC-TMT

One interesting advantage of pSILAC-DIA over both pSILAC-MS1 and pSILAC-TMT is the availability of multiple quantitative data points. This is because, for a given peptide (an H/L pair), very limited quantitative features can be obtained for pSILAC-MS1 (that are based on the precursor pair) or pSILAC-TMT (that are based on the reporter ion ratio from those identified MS2 or SPS-MS3 scan event). However, in pSILAC-DIA, all the high-resolution fragment ions of the peptide can be used for quantifying H/L ratios. Although normally the filtered top 3-6 fragment ions are already enough to determine the ratio, potentially almost all fragment ions can be used as replicated information, yielding a more robust estimation of H/L ratio. Please see **Appendix Figures S5** and **S6** for the further illustration. Although not shown in the current study, more ions could also potentially facilitate the turnover calculation for different peptidoforms (i.e., the same peptide sequences with different post-translational modifications and modification combinations).

pSILAC-DIA vs pSILAC-TMT

The hyper-multiplexing approach combining "pSILAC" and "MS2-tag based quantification" is not actually new, and has been already proposed in 2010 in which pSILAC labeling was combined with iTRAQ quantification (i.e., pSILAC-iTRAQ) (Jayapal et al, 2010; Hinkson & Elias, 2011). Recently, pSILAC-TMT has emerged as a powerful MS based tool to measure protein turnover (Welle et al, 2016; Savitski et al, 2018; Zecha et al, 2018), especially because the SPS-MS3 quantification was shown to effectively reduce co-isolation issue in TMT measurements (McAlister et al, 2014; Zecha et al, 2018). Furthermore, there are more quantitative channels from TMT tags and better MS instruments available supporting high-resolution measurements needed for TMT quantification. Indeed, the multiplexity of pSILAC-TMT (Welle et al, 2016; Zecha et al, 2018) impairs the missing value problem and decreases the number of sample injections required for a pSILAC experiment.

However, in our hands pSILAC-DIA achieved comparable proteome coverage to pSILAC-TMT with a similar total MS measurement time. This could be due to the facts that e.g., there is no fractionation needed in pSILAC-DIA and that extra time for MS2 scans (that are not used for quantitation) was spent during SPS-MS3 TMT analysis. The comparable coverage between DIA and TMT approaches is consistent to a previous report comparing the performance of the two (Muntel et al, 2019). Besides the reduced ion complexity and

more quantitative data points mentioned above (**Appendix Figures S2**, **S5,** and **S6**), in particular, we also summarize other advantages of pSILAC-DIA over pSILAC-TMT below. **Therefore, we suggest all these considerations together make pSILAC-DIA a competitive, *if not a better,* method than pSILAC-TMT.**

1) Even the current most optimized version of SPS-MS3 based pSILAC-TMT seems to (only) provide a similar quantitation performance of turnover rates to pSILAC-MS1, but not better. This may be not surprising as SPS-MS3 only largely reduces the co-isolation problem but does not totally eliminate it (Muntel et al, 2019), which stems from the TMT design. Thus, in the previous publications, the pSILAC-TMT (MS2) generated less-precise turnover rates than pSILAC-MS1 (Welle et al, 2016), whereas the pSILAC-TMT (SPS-MS3) with a further bioinformatic correction dealing with the normalization bias (Zecha et al, 2018), makes the approach become close or comparable to pSILAC-MS1 results in precisely determining the H/L ratios and thus, k_{loss}.

   In contrast, again, with **Appendix Figures S3** and **S4**, we show compelling results that pSILAC-DIA can generate significantly better quantification accuracy and precision than pSILAC-MS1 (see above).

2) The problem with large-scale, multi-batch TMT data was recently reported by experienced labs (Brenes et al, 2019), suggesting the pSILAC-TMT approach might not be ready to be used by every lab, and importantly, to be used to compare protein turnover across multiple cell lines (like our application shown in this study) and conditions. Indeed, false positives, batch effects, missing values, and other issues were reported in a multi-batch TMT experiment despite the use of SPS-MS3 (Brenes et al, 2019).

3) Experimental design flexibility. In a pSILAC experiment, a 10-time point sampling during pulse-chase process does not have to be always necessary (10 TMT channels were used in (Zecha et al, 2018)). More importantly, the pSILAC-DIA offers a greater flexibility in trouble-shooting a proteomic experiment (e.g., if one of the samples has some technical or experimental problem, in pSILAC-DIA workflow the problematic sample can be easily re-processed and measured with new data combined, whereas in pSILAC-TMT the whole sample set has to be re-measured).

4) Machine flexibility. Currently, the SPS-MS3 seems to be essential for pSILAC-TMT to ensure the quality of turnover estimation to be comparable to pSILAC-MS1 (Zecha et al, 2018). This function, however, is only provided in Fusion-type Orbitrap analyzers from Thermo Scientific. pSILAC-DIA has a much broader application potential in this regard.

5) pSILAC-DIA might request lower cost for proteomics labs, considering the cost of TMT reagents for lots of samples.

6) Potential of pSILAC-DIA in analyzing peptide PTM isoforms (peptidoforms). DIA-MS includes extra information of the perfect co-elution behavior between all fragment ions of the same peptide isoform

along the liquid chromatography. This feature will offer much higher ability in discriminating SILAC H/L ratios for discriminating PTM isoforms (Rosenberger et al, 2017). Instead, in pSILAC-TMT, those different PTM isoforms of the same peptide backbone eluting together (or, arising from other pSILAC channels of a different time point) can be easily co-isolated for MS2 and SPS-MS3 analysis, and thus, interfere with each other.

7) Potential of analyzing „heavy“ and „light“ signals separately for non-steady state measurement. Although for the protein turnover measurement a general assumption is applied that cells are growing in the **steady state** (i.e., the sum of „heavy“ and „light“ signals is stable during labeling), DIA data provides the possibility to directly and robustly analyze „heavy“ and „light“ version of proteins separately. This ability will be useful for studying dynamic processes e.g., systems under a perturbation. To achieve such a task, pSILAC-TMT analysis would have to rely on a complex statistical approach (Savitski et al, 2018).

Supplementary Note 4: Comparison between the relative isotope abundance (RIA)-based and normalized light intensities (NLI)-based k_{loss} calculations

The RIA algorithm used in this study determines the protein turnover rate in the steady state considering both “light” (L) and “heavy” (H) intensities (see **Methods**). However, the high accuracy of our DIA-MS measurement actually enables measuring L and H peptide intensities separately in the pSILAC experiment. A simpler approach to determine a *de facto* protein degradation rate would be to directly calculate the rate of loss from the L (*i.e.*, unlabeled peptide) intensities. Therefore, in this Supplementary Note, we performed such a calculation termed normalized light intensity (NLI)-based method (see **Methods**) and compared the results to results determined by the RIA algorithm.

Regarding the NLI calculation process, firstly, as in all label-free quantification experiments, we normalized the total identified DIA signals based on the sum of the heavy and light channels across time. Then, we extracted the light channel quantities (which showed a perfect pattern of gradual loss of the light intensity over time) by fitting the desired curve on each peptide. Finally, we aggregated peptide precursor k_{loss} values to the protein AS isoform groups level. For the purpose of comparison, the calculation was performed for the same data set (twelve HeLa cell lines), using the same Spectronaut results as an input for the algorithm, and the same parameters for peptide k_{loss} averaging as in the case of the RIA-based data presented in our study.

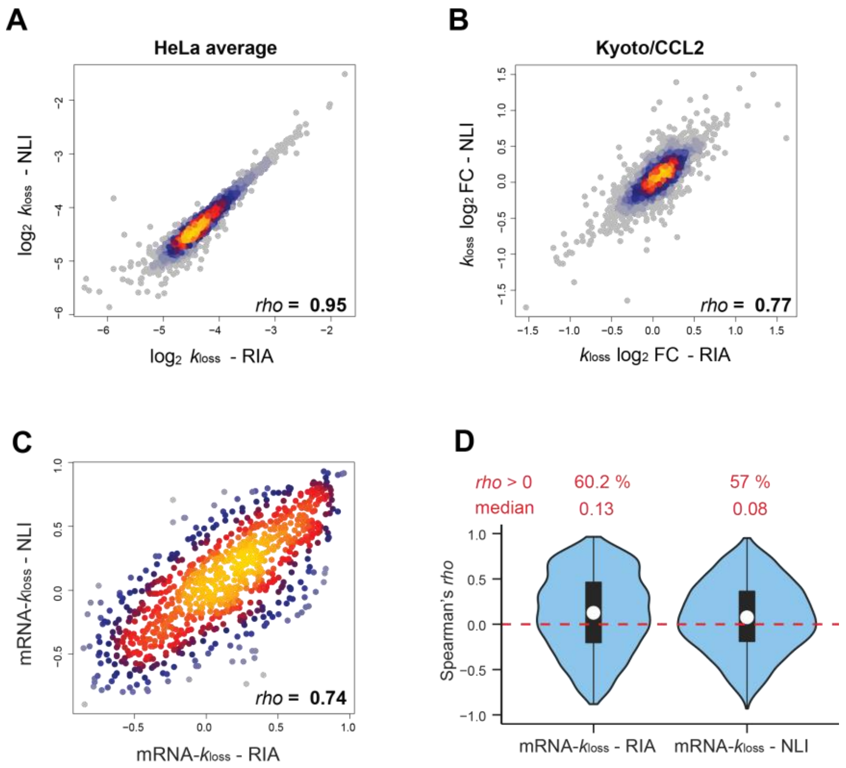

Figure 5: Correlation between RIA- and NLI-based log₂ k_{loss} and corresponding mRNA-k_{loss} values*. Correlation between $\log_2 k_{loss}$ absolute values (average of all HeLa cell lines; **A**) and relative values (six HeLa Kyoto cell lines/ six HeLa CCL2 cell lines log2 fold change; **B**). Correlation between the across-cell lines protein AS isoform-specific mRNA-k_{loss} correlation **(C)** and corresponding mRNA-k_{loss} distributions **(D)**. Rho indicates Spearman’s correlation.*

We first assessed whether the RIA and NLI methods provided comparable results. The $\log_2$ scale k_{loss} correlation analysis based on averaged value of all HeLa cells demonstrated a high correlation (*rho* = 0.95; **Figure 5A**). The relative correlation analysis between HeLa Kyoto and CCL2 samples also revealed that consistent results were largely achieved between RIA and NLI (*rho* = 0.77; **Figure 5B**). This highlights the possibility of directly using light signals in a two-channel pSILAC-DIA experiment for k_{loss} calculation. Next, we calculated the proteome-wide, protein AS isoform-specific mRNA-k_{loss} correlation between all twelve HeLa cells using NLI-derived k_{loss} values. We found that despite this process involved independent calculation in each of twelve HeLa cells, the correlation coefficients still significantly correlated (**Figure 5C**), which is as strong as the relative ratio-based correlation. Finally, we assessed whether majority of the NLI-derived mRNA-k_{loss} *rho* remained positive (i.e., above 0; **Figure 5D**). Indeed, although NLI generated a lower median (0.08, compared to 0.13 in RIA), 57% of the values were still above 0. This result thus supports the notion that the cells are likely to use protein degradation to fine-tune mRNA variation.

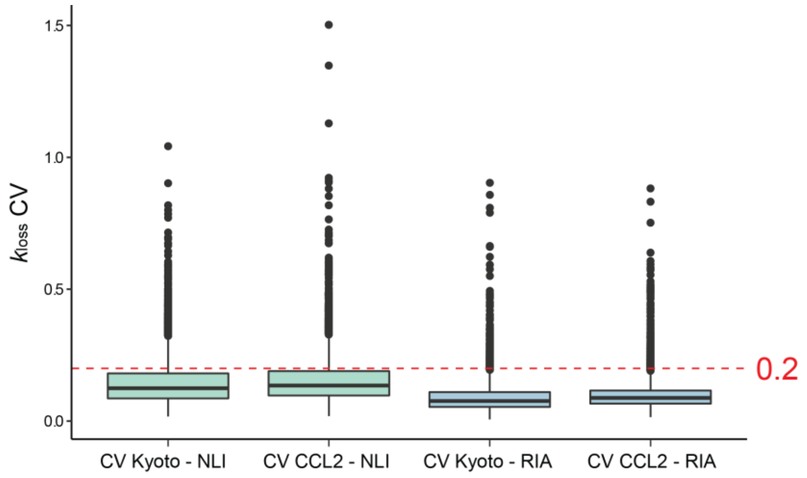

Figure 6: Comparison of RIA- and NLI-based k_{loss} precision across the HeLa cell lines. *Coefficient of variation (CV) was calculated for each protein AS isoform group within six HeLa Kyoto or six HeLa CCL2 cell lines. A threshold of CV < 0.2 (red line) was highlighted.*

We next asked whether NLI derives precise measurement of k_{loss} similar to RIA results. We performed a comparison of CVs of protein-level k_{loss} values generated by both methods, across replicates of CCL2 and Kyoto cells. Interestingly, we found RIA still generated more precise quantification with a statistical significance (Wilcoxon test, $p < 1 \times 10^{-16}$ for both CCL2 and Kyoto HeLa cells; **Figure 6**). This seems to suggest the calculation of k_{loss} using ratio of H and L signals (i.e. RIA) is still more stable than single L channel-based determination (i.e., NLI), possibly because the decrease of light signals is small in early time points (e.g., at 1 hour or 4.5 hours), which essentially presents a challenging case of relative quantification even for DIA-MS.

Following, we repeated the analysis presented in **Figure 3D-G** (main text in the article), but limited to k_{loss} values with a CV < 0.2 for both RIA and NLI, to achieve a fair comparison. The results

were consistent to **Figure 3D-G** in the main text. As shown in **Figure 7**, the absolute mRNA-k_{loss} correlation was determined to be ρ = -0.22 by RIA and -0.26 by NLI (**Figure 7A&B**; n = 2,513; $p <$ 0.0001) in all protein AS isoform groups (including both UQ and SM), and the absolute protein-k_{loss} correlation was even more negative (ρ = -0.40 by RIA and -0.42 by NLI; n = 2,513; $p < 0.0001$). Thus, the degradation seems to be slower for genes with higher expression on the absolute scale. However, when the Kyoto/CCL2 fold changes are analyzed, slight but significant positive across-gene mRNA-k_{loss} correlations were obtained, which is identical to **Figure 3** in the main text (**Figure 7C&D**; ρ = 0.13 for RIA, ρ = 0.09 for NLI; n = 2,513; $p < 0.0001$). Altogether, NLI and RIA were in a good agreement about absolute and relative correlations between mRNA and k_{loss}.

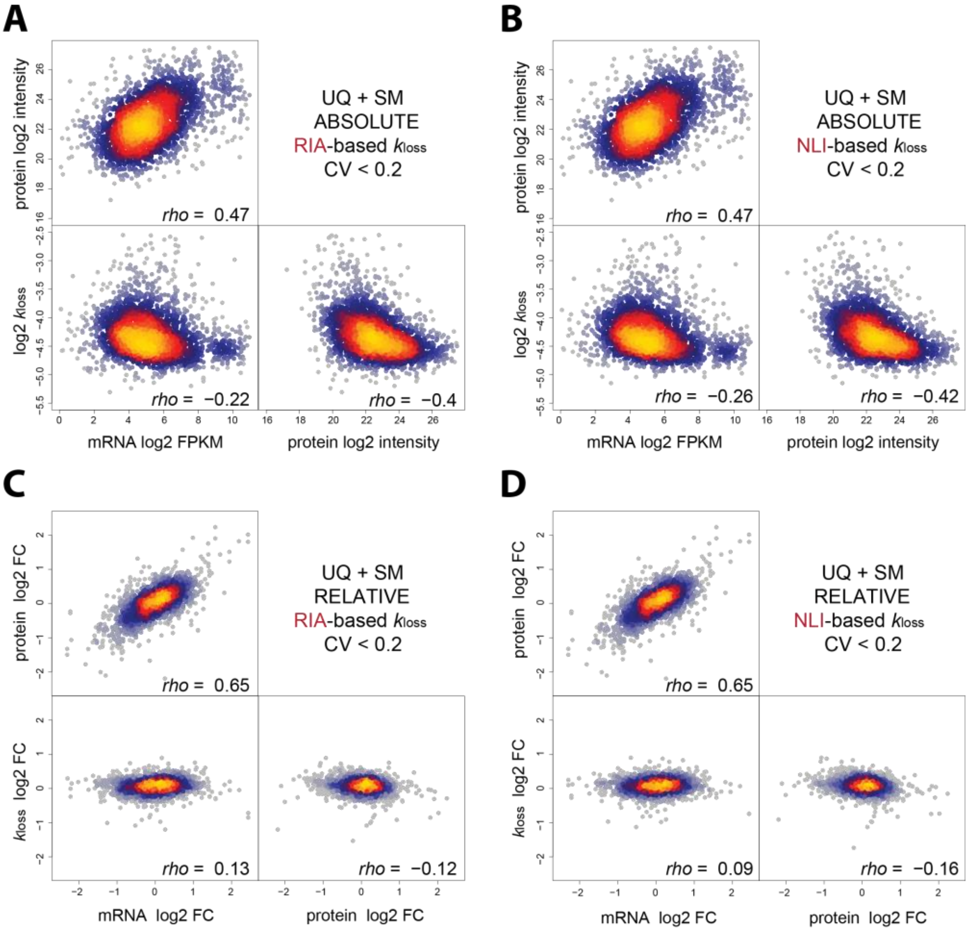

Figure 7: Absolute and relative correlation analysis using RIA- and NLI-based k_{loss} values. (A-B) *Across-gene Spearman's absolute correlation between indicated values (average from all cell lines).* ***(C-D)*** *Spearman's correlation between indicated values using relative quantification data (Kyoto/CCL2 fold change).*

Finally, we assessed the overall protein buffering significance in the NLI- and RIA-based data (Kyoto and CCL2 CV < 0.2 in both) to confirm whether the conclusions remain the same when k_{loss} is directly derived from the loss of the light, unlabeled, peptide. According to **Figure 3H** in the main text of the manuscript, we assessed the mRNA-protein and the mRNA-k_{loss} correlations for protein AS isoform groups (including both UQ and SM) that were either differentially expressed (t-test, Benjamini-

Hochberg FDR < 0.01) between Kyoto and CCL2, or not (**Figure 8A**). Consistently with **Figure 3H** in the main text, we observed for both k_{loss} calculation methods proteins significantly regulated between the cell lines show a significantly lower mRNA-k_{loss} correlation than proteins which were not differentially expressed.

In **Figure 8B**, we provide mRNA-k_{loss} correlation for both methods distributed to protein complex participation (i.e., according to CORUM annotation, proteins known to be the subunits of a stable protein complex are marked as "Complex IN", and proteins without a protein complex annotation are marked as "Complex OUT"). Because protein complex stoichiometry is a general mechanism that is well known for protein buffering, this analysis confirms mRNA-k_{loss} correlation is indeed a good measure. The better mRNA-k_{loss} correlation tend to result in more constraints at the protein level and thus worse mRNA-protein correlations (**Figure 8B**). Compellingly and conceivably, proteome wide mRNA-k_{loss} is a good indicator of protein abundance buffering mechanism independently on the method of k_{loss} calculation.

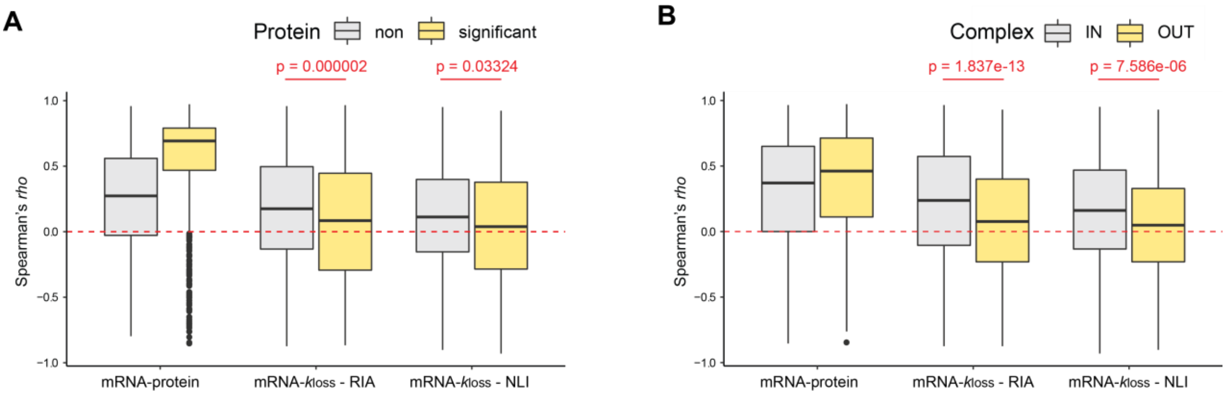

Figure 8: *RIA- and NLI-based mRNA-k_{loss} protein AS isoform group-specific correlation resolved to* ***(A)*** *proteins differentially expressed between the cell line or not and* ***(B)*** *protein complex subunits and other proteins.*

Supplementary Methods

Comparison between MS1 and MS2- based SILAC quantification

For the analysis presented in **Figures S2-S6**, the same data analysis workflow was used as for the data described in the Methods section in the main text. Briefly, the same library (i.e., a hybrid library containing 159,963 peptide precursors corresponding to 11,623 proteins, including the Top 6 fragment ions per peptide and excluding the b-type ions), the same FASTA protein sequence database, and workflow (i.e., the Inverted Spike-In workflow) were used for targeted data extraction of three raw files from HeLa 7 (time points 1, 4.5, and 11 hours). The interference correction was applied to both MS1- and MS2- based quantification with minimum of two values for MS1 quantification (the mono-isotopic precursor and isotopic envelope) and a minimum of three fragment ions for MS2 quantification.

For fragment ion-based ratio quantification, the fragment ion-level report was generated in Spectronaut, and the SILAC H/L ratios were further calculated, processed, and visualized in R. The ratios were calculated using the matched „light“ and „heavy“ fragment ion intensities (F.PeakArea). The F.ExcludedFromQuantification column was used to exclude potentially interfering ions that were filtered using the interference filtering. F.Charge column was used to report the fragment ion charge state.

For peptide-precursor MS-1 and MS2-based quantification, the fragment group-level report was generated in Spectronaut, and the ratios were further calculated, processed, and visualized in R. The ratios were calculated using the matched „light“ and „heavy“ (FG.IsotopeLabelType) fragment ion group intensities for MS1- (FG.MS1Quantity) and MS2-based quantification (FG.MS2Quantity). Thus, we used all peptide precursors quantified by Spectronaut with no additional quantification filtering applied. FG.Charge column was used to report the fragment ion charge state.

Thus, in the analyses described below, we compared four data sets below, each one of them containing HeLa 7 H/L ratio quantification in three labeling time points.

1) Top 6 – this data set contained all H/L ratios computed using individual fragment ions, with no interference filtering applied.
2) Top 6 + interference filtering – this data set contained all H/L ratios computed using individual fragment ions; both „light“ and „heavy“ fragment ions were required to pass the interference filtering.
3) MS2 – this data set comprised peptide precursor H/L ratios quantified using MS2-based quantification in Spectronaut (i.e., the sum fragment ions after interference filtering).
4) MS1 – this data set comprised peptide precursor H/L ratios quantified using MS1-based quantification in Spectronaut (i.e., the sum of min two MS-1 signals after interference filtering).

These data sets were used for the following analyses:

1) All H/L ratios of fragment ions/peptide precursors distribution visualization across different labeling time points.
2) Protein H/L ratio summary: for each protein AS group a median protein ratio and a standard deviation were calculated using all values (fragment ions/peptide precursors H/L ratios) assigned to a protein AS group. Furthermore, a correlation (Spearman's rank correlation) between all SILAC „light" and „heavy" intensities for every time point was calculated for each protein group.
3) For every protein AS group, we created a visualization showing correlation between „light" and „heavy" channels in three different time points, and a boxplot summarizing corresponding heavy/light ratios. Selected examples from these analyses are presented in the figures.

Comparison of within- and between- protein alternative splicing (AS) groups peptide profile k_{loss} variability

In **Figure 4**, certain protein AS isoforms of the same gene may have differential degradation rates or get differentially degraded between Kyoto and CCL2 cell lines. We plotted the across-cell line variability of peptide profiles within genes, within protein AS isoform groups and between protein AS groups of the same gene in additional analyses (shown in **Figure S7**).

1) Using the peptide level k_{loss} data, we estimated a standard deviation of all peptide $\log_2 k_{\text{loss}}$ values assigned to each gene (n = 2,390, $n_{\text{peptides}} > 1$) and to each protein AS group (n = 3,848, $n_{\text{peptides}} > 1$). We further visualized distributions of the standard deviations for genes and protein AS groups, and using Wilcoxon test, we tested whether the observed difference was statistically significant. This analysis was performed separately for the HeLa 1 $\log_2 k_{\text{loss}}$ value, for HeLa Kyoto average $\log_2 k_{\text{loss}}$ value, and for the HeLa CCL2 average $\log_2 k_{\text{loss}}$ value.
2) Similar to a gene/protein-specific correlation between different layers, we calculated a correlation coefficient (Spearman's *rho*) between peptide $\log_2 k_{\text{loss}}$ profiles across all HeLa cell lines. For every protein AS group (with at least two peptides quantified, n = 3,848), the correlations for all binary peptide comparisons were calculated (i.e., within- protein AS groups). For every gene (with two to four protein AS groups quantified with at least two peptides, n = 1,477), the correlations were calculated for all binary peptide comparison between peptides corresponding to different AS isoforms of the same gene (i.e., between-protein AS groups). We then visualized the distribution of all within- and between- protein AS groups correlations, and estimated statistical significance of the difference using Wilcox test. The same analysis was performed for a subset of 30 genes and the

corresponding 60 protein AS groups for which we reported differential degradation between HeLa CCL2 and Kyoto ($p < 0.05$, only genes with 2 protein AS groups were used for this analysis).

Supplementary Figures Captions

Appendix Figure S1: **Principles and assumptions underlying protein degradation quantification using pSILAC-DIA**. **(A)** Routine design of a simple, dynamic SILAC labeling experiment. Steady-state cells are grown in a culturing medium containing the „light" versions of lysine and arginine. At time_0, the medium is exchanged for a cell culture medium containing the "„heavy"" versions of the amino acids ("Arg10" and "Lys8"). The cells are then harvested at several different time points, when different fraction of the proteome is increasingly labeled by the "„heavy"" amino acids. Under the hypothesis of "steady-state", the seeding density and the havest time points must be carefully selected to ensure exponential proliferation of the cells during the whole time course of the experiment to maintain the proliferation rate. **(B)** DIA-MS data of a pSILAC experiment. The „light" and „heavy" peptide precursors (MS1) and fragment ions (MS2) co-elute, but can be distinguished by a mass shift (m/z) due to the SILAC labeling. The „light" and „heavy" channels are aligned by Spectronaut and can be accurately quantified by setting the same peak boundaries for „light" and „heavy" ions, further facilitating the detection of the low intensity „heavy" signal in the early time points by the Inverted Spike-In workflow. **(C)** k_{loss} estimation. At different time points, a different fraction of the proteome is present in the unlabeled state (L) or in the labeled state (H). Again, as the cells are maintained in a steady-state, the total protein concentration (i.e., L+H) does not change over the time course of the experiment. The „light" peptide fraction (RIA) at time point t, is then calculated for each time point, and these values are used to perform a fit by the nonlinear least squares algorithm. Please also refer to the **Methods** section for experimental details.

Appendix Figure S2: **Charge state distribution of the MS1- and MS2-based pSILAC-DIA quantification**. Charge distribution of **(A)** peptide precursors and **(B)** fragment ions in the example of time point T1 of the HeLa 7 data set. **(C)** A Table summarizing the theoretical mass difference between „light" and „heavy" versions of lysine- and arginine-containing ions at different charge states indicating the difficulty for isotopic recognition.

Appendix Figure S3: **Comparison between MS1-based and MS2-based quantification of peptide precursor SILAC H/L ratios**. **(A)** Distribution of all peptide-precursor H/L ratios quantified in each time point using pSILAC-DIA in HeLa 7 (n = 159,945 in both MS1 and MS2). The ratios were calculated using the matched „light" and „heavy" (FG.IsotopeLabelType) fragment ion group intensities from MS1- (FG.MS1Quantity) and MS2-based quantification (FG.MS2Quantity). The numbers indicate standard deviations of the $\log_2$ H/L ratio distribution in each time point. **(B)** Distribution of protein $\log_2$ H/L ratio standard deviations. For each protein group a standard deviation was calculated using all values (peptide-precursor $\log_2$ H/L ratios) assigned to a protein AS group. **(C)** Correlation between „heavy" and „light" SILAC intensities. Spearman's rank correlation between all

„light“ and „heavy“ peptide-precursors quantified within each time point was calculated for each protein AS group.

Appendix Figure S4: **Comparison between MS1-based and MS2-based quantification of peptide precursor ratios using pSILAC-DIA.** Similar to Figure 2C-F, for the selected example proteins in **(A-C),** the scatter plots show the correlation between SILAC „light“ and „heavy“ peptide-precursor intensities quantified in either MS1 or MS2 from the example data in Figure S3; a linear regression line (dashed) was added for each time point indicated by different colors. The boxplots summarize the corresponding heavy/light ratios within each time point. The numbers of peptide precursors (n) in each time point were 75, 821, and 115, for **(A)**, **(B)**, and **(C)**, respectively.

Appendix Figure S5: **MS2-based SILAC H/L ratios quantified in pSILAC-DIA**. For the selected example proteins in **(A-B)**, the scatter plots show the correlation between Top 6 SILAC „light“ and „heavy“ fragment ions, between Top 6 „light“ and „heavy“ fragment ions that passed the interference filtering step, and between „light“ and „heavy“ peptide precursors quantified using the MS2 quantification (i.e., the sum of the Top 6 fragment ions that passed the interference filtering). A linear regression line was added for each time point as indicated by different colors; the numbers indicate the number of values in each time point. **(C-D)** The boxplots summarize the corresponding H/L ratios within each time point. The width of the boxes is scaled with the number of values.

Appendix Figure S6: **Visualization of „light“ and „heavy“ MS2 and MS1 quantitative signals using the “XIC graph”**. **(A)** All fragment ions that can be potentially used for pSILAC-DIA quantification. For this figure exclusively, no fragment ion filtering was applied during the library generation step whereas the b-type ions were removed. **(B)** Only the Top 6 fragment ions are depicted for the same peptide precursor. **(C)** The MS1-XIC of the selected peptide precursor. In **(A-C)**, dashed lines mark the potentially interfering ions that are then removed from quantification, while solid lines mark the ions that passed the interference filtering. The graphics above the zero line shows the light-channel XICs, while the heavy-channel XICs are shown below the zero line. The vertical lines separate the XICs of three pulse labeling time points.

Appendix Figure S7: Peptide k_{loss} variability analysis within- and between- protein alternative splicing (AS) groups. (A) Distribution of all gene and protein AS group $\log_2 k_{\text{loss}}$ standard deviations. The standard deviations were estimated using all peptide $\log_2 k_{\text{loss}}$ values assigned to either a gene (n = 2,390) or a protein AS group (n = 3,848). **(B)** Correlation (Spearman’s *rho*) between peptide $\log_2 k_{\text{loss}}$ profiles across HeLa cell lines. For every protein AS group (with at least two peptides quantified, n = 3,848), the correlations for all binary peptide comparisons were calculated (i.e., within- protein AS groups). For every gene (with two to four protein AS groups quantified with at least two peptides, n = 1,477), the correlations were calculated for all binary peptide comparison between peptides

corresponding to different AS isoforms of the same gene (i.e., between- protein AS groups). **(C)** The same analysis was performed for a subset of 30 genes and the corresponding 60 protein AS groups for which we previously reported differential degradation between HeLa CCL2 and Kyoto. *p* values were estimated using the Wilcox test.

Appendix Figure S8: Intensity distributions of protein and mRNA measurements corresponding to mRNA-k_{loss} quintiles. Log_2 protein intensities **(A)** and mRNA intensities **(B)** of protein AS isoform groups included in mRNA-k_{loss} quintiles shown in **Figure 5D.**

References

Albert FW, Muzzey D, Weissman JS, Kruglyak L (2014) Genetic influences on translation in yeast. *PLoS Genet* **10:** e1004692

Bader DM, Wilkening S, Lin G, Tekkedil MM, Dietrich K, Steinmetz LM, Gagneur J (2015) Negative feedback buffers effects of regulatory variants. *Molecular systems biology* **11:** 785

Brenes A, Hukelmann J, Bensaddek D, Lamond AI (2019) Multibatch TMT Reveals False Positives, Batch Effects and Missing Values. *Molecular & cellular proteomics : MCP* **18:** 1967-1980

Claydon AJ, Beynon R (2012) Proteome dynamics: revisiting turnover with a global perspective. *Mol Cell Proteomics* **11:** 1551-1565

Franken H, Mathieson T, Childs D, Sweetman GM, Werner T, Togel I, Doce C, Gade S, Bantscheff M, Drewes G, Reinhard FB, Huber W, Savitski MM (2015) Thermal proteome profiling for unbiased identification of direct and indirect drug targets using multiplexed quantitative mass spectrometry. *Nature protocols* **10:** 1567-1593

Hinkson IV, Elias JE (2011) The dynamic state of protein turnover: It's about time. *Trends Cell Biol* **21:** 293-303

Jayapal KP, Sui S, Philp RJ, Kok YJ, Yap MG, Griffin TJ, Hu WS (2010) Multitagging proteomic strategy to estimate protein turnover rates in dynamic systems. *Journal of proteome research* **9:** 2087-2097

Liu Y, Borel C, Li L, Muller T, Williams EG, Germain PL, Buljan M, Sajic T, Boersema PJ, Shao W, Faini M, Testa G, Beyer A, Antonarakis SE, Aebersold R (2017) Systematic proteome and proteostasis profiling in human Trisomy 21 fibroblast cells. *Nature communications* **8:** 1212

Liu Y, Mi Y, Mueller T, Kreibich S, Williams EG, Van Drogen A, Borel C, Frank M, Germain PL, Bludau I, Mehnert M, Seifert M, Emmenlauer M, Sorg I, Bezrukov F, Bena FS, Zhou H, Dehio C, Testa G, Saez-Rodriguez J et al (2019) Multi-omic measurements of heterogeneity in HeLa cells across laboratories. *Nat Biotechnol* **37:** 314-322

Mathieson T, Franken H, Kosinski J, Kurzawa N, Zinn N, Sweetman G, Poeckel D, Ratnu VS, Schramm M, Becher I, Steidel M, Noh KM, Bergamini G, Beck M, Bantscheff M, Savitski MM (2018) Systematic analysis of protein turnover in primary cells. *Nature communications* **9:** 689

McAlister GC, Nusinow DP, Jedrychowski MP, Wuhr M, Huttlin EL, Erickson BK, Rad R, Haas W, Gygi SP (2014) MultiNotch MS3 enables accurate, sensitive, and multiplexed detection of differential expression across cancer cell line proteomes. *Anal Chem* **86:** 7150-7158

Muntel J, Kirkpatrick J, Bruderer R, Huang T, Vitek O, Ori A, Reiter L (2019) Comparison of Protein Quantification in a Complex Background by DIA and TMT Workflows with Fixed Instrument Time. *J Proteome Res* **18:** 1340-1351

Rosenberger G, Liu Y, Rost HL, Ludwig C, Buil A, Bensimon A, Soste M, Spector TD, Dermitzakis ET, Collins BC, Malmstrom L, Aebersold R (2017) Inference and quantification of peptidoforms in large sample cohorts by SWATH-MS. *Nat Biotechnol* **35:** 781-788

Rost HL, Liu Y, D'Agostino G, Zanella M, Navarro P, Rosenberger G, Collins BC, Gillet L, Testa G, Malmstrom L, Aebersold R (2016) TRIC: an automated alignment strategy for reproducible protein quantification in targeted proteomics. *Nat Methods* **13:** 777-783

Savitski MM, Zinn N, Faelth-Savitski M, Poeckel D, Gade S, Becher I, Muelbaier M, Wagner AJ, Strohmer K, Werner T, Melchert S, Petretich M, Rutkowska A, Vappiani J, Franken H, Steidel M, Sweetman GM, Gilan O, Lam EYN, Dawson MA et al (2018) Multiplexed Proteome Dynamics Profiling Reveals Mechanisms Controlling Protein Homeostasis. *Cell* **173:** 260-274 e225

Welle KA, Zhang T, Hryhorenko JR, Shen S, Qu J, Ghaemmaghami S (2016) Time-resolved Analysis of Proteome Dynamics by Tandem Mass Tags and Stable Isotope Labeling in Cell Culture (TMT-SILAC) Hyperplexing. *Mol Cell Proteomics* **15:** 3551-3563

Zecha J, Meng C, Zolg DP, Samaras P, Wilhelm M, Kuster B (2018) Peptide Level Turnover Measurements Enable the Study of Proteoform Dynamics. *Mol Cell Proteomics* **17:** 974-992

Figure S1

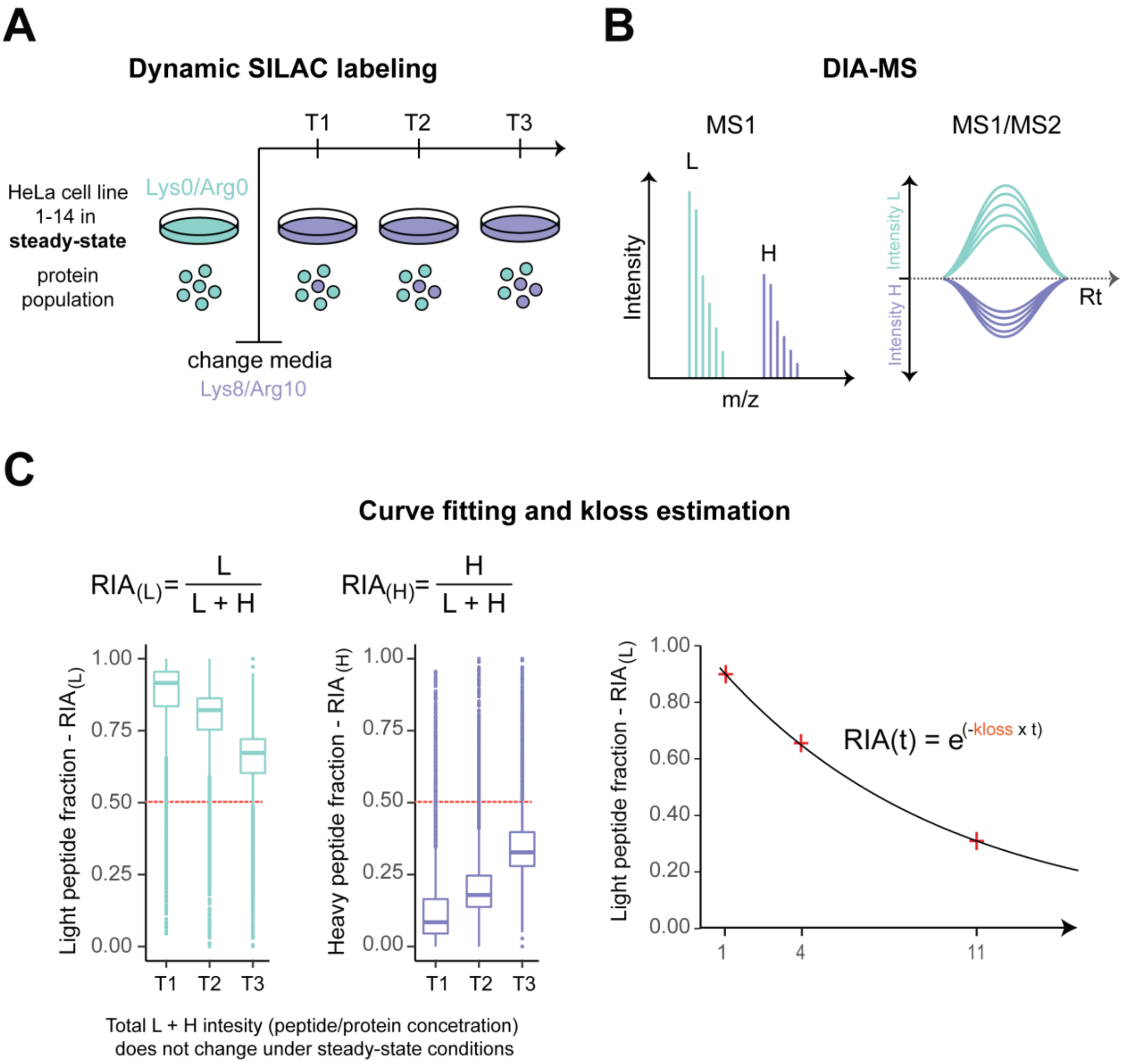

A
Dynamic SILAC labeling
T1
T2
T3
HeLa cell line
1-14 in
steady-state
protein
population
Lys0/Arg0
change media
Lys8/Arg10
B
DIA-MS
MS1
MS1/MS2
L
H
Intensity
m/z
Intensity L
Intensity H
Rt
C
Curve fitting and kloss estimation
RIA(L)= L / L + H
RIA(H)= H / L + H
Light peptide fraction - RIA(L)
1.00
0.75
0.50
0.25
0.00
T1
T2
T3
Heavy peptide fraction - RIA(H)
1.00
0.75
0.50
0.25
0.00
T1
T2
T3
Light peptide fraction - RIA(L)
1.00
0.80
0.60
0.40
0.20
0.00
1
4
11
RIA(t) = e(-kloss x t)
Total L + H intesity (peptide/protein concetration)
does not change under steady-state conditions

Figure S2

A

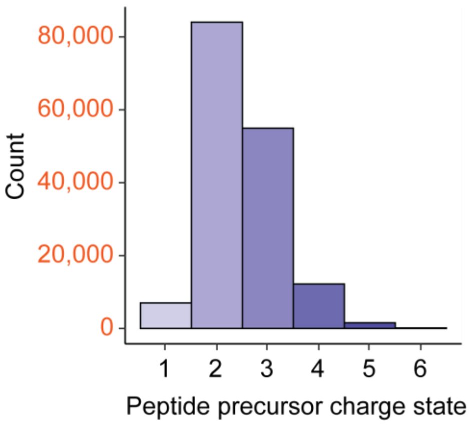

B

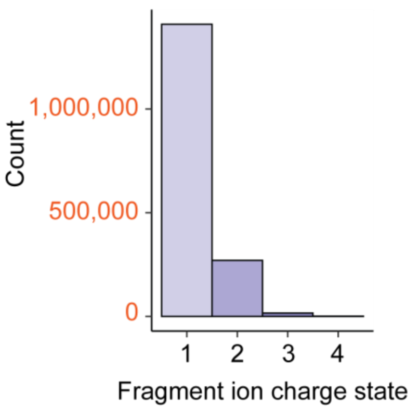

C

Charge	K8 - K0	R10 - R0
1	8.014	10.008
2	4.007	5.004
3	2.671	3.336
4	2.004	2.502
5	1.603	2.002
6	1.336	1.668

Figure S3

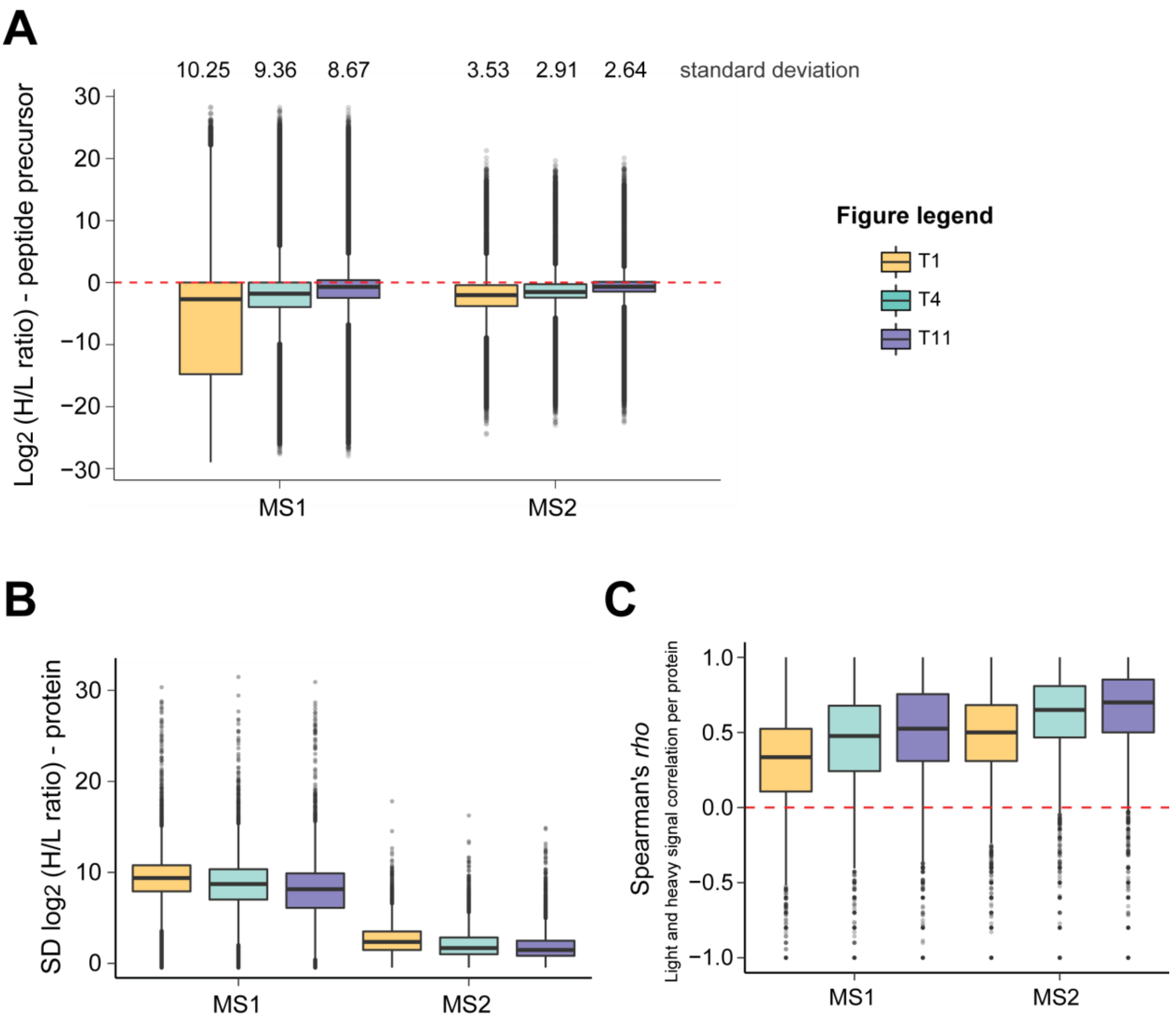

A
10.25
9.36
8.67
3.53
2.91
2.64
standard deviation
Log2 (H/L ratio) - peptide precursor
30
20
10
0
−10
−20
−30
MS1
MS2
Figure legend
T1
T4
T11
B
SD log2 (H/L ratio) - protein
30
20
10
0
MS1
MS2
C
Spearman's rho
Light and heavy signal correlation per protein
1.0
0.5
0.0
−0.5
−1.0
MS1
MS2

Figure S4

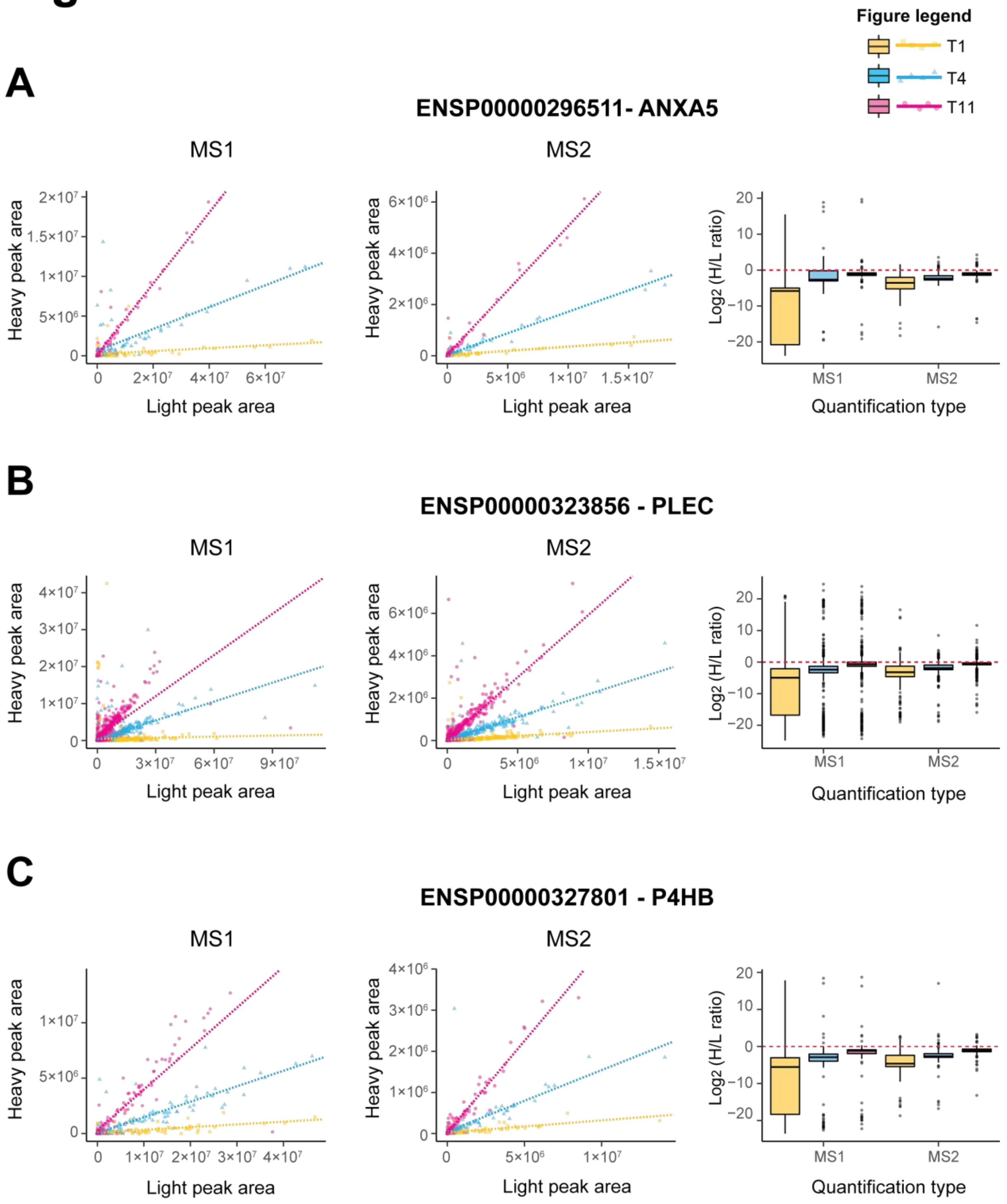
Figure legend
T1
T4
T11
A
ENSP00000296511- ANXA5
MS1
MS2
Heavy peak area
Light peak area
Log2 (H/L ratio)
Quantification type
B
ENSP00000323856 - PLEC
MS1
MS2
Heavy peak area
Light peak area
Log2 (H/L ratio)
Quantification type
C
ENSP00000327801 - P4HB
MS1
MS2
Heavy peak area
Light peak area
Log2 (H/L ratio)
Quantification type

Figure S5

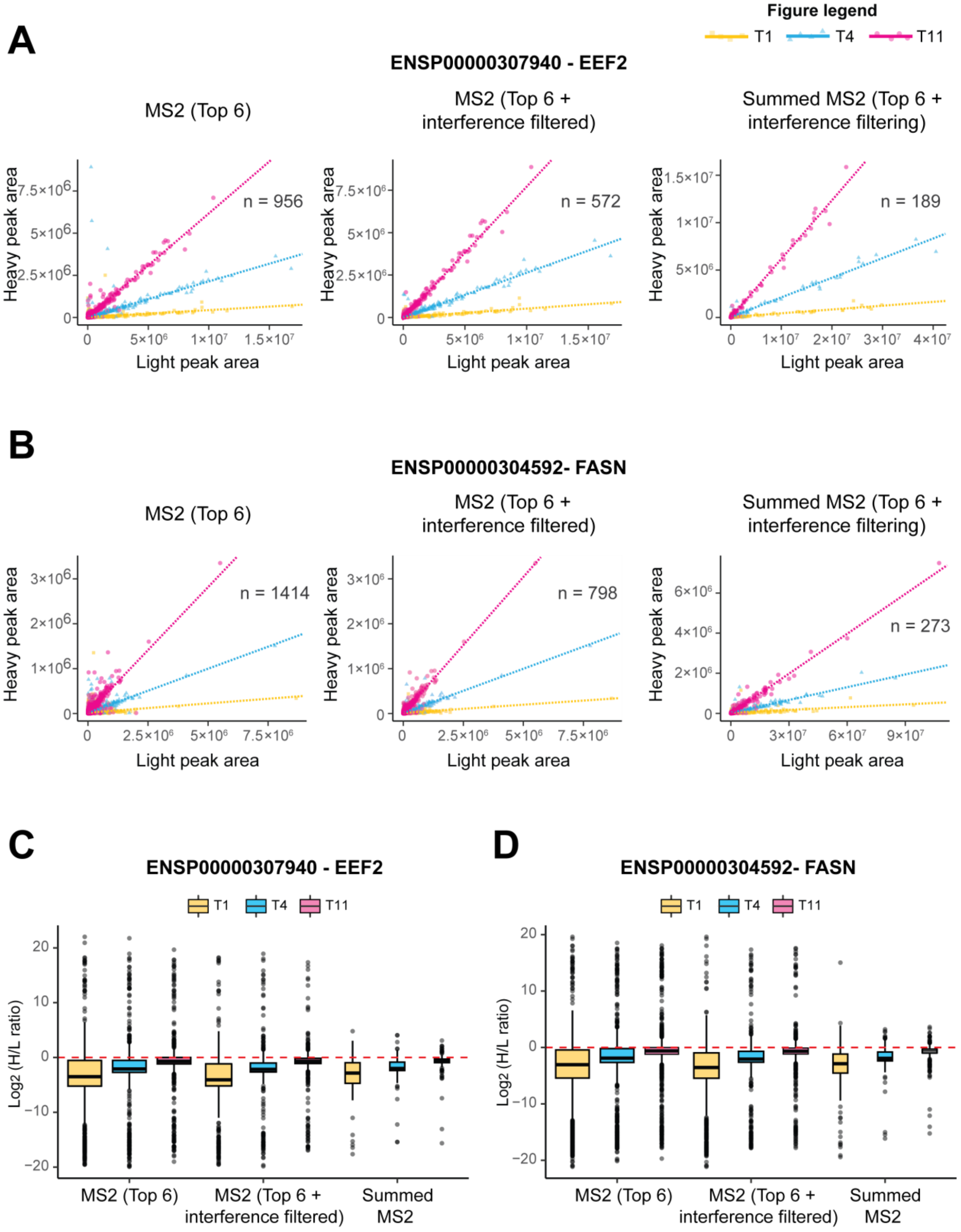
Figure legend
T1
T4
T11
A
ENSP00000307940 - EEF2
MS2 (Top 6)
MS2 (Top 6 + interference filtered)
Summed MS2 (Top 6 + interference filtering)
n = 956
n = 572
n = 189
Heavy peak area
Light peak area
B
ENSP00000304592- FASN
MS2 (Top 6)
MS2 (Top 6 + interference filtered)
Summed MS2 (Top 6 + interference filtering)
n = 1414
n = 798
n = 273
Heavy peak area
Light peak area
C
ENSP00000307940 - EEF2
D
ENSP00000304592- FASN
Log2 (H/L ratio)
MS2 (Top 6)
MS2 (Top 6 + interference filtered)
Summed MS2

Figure S6

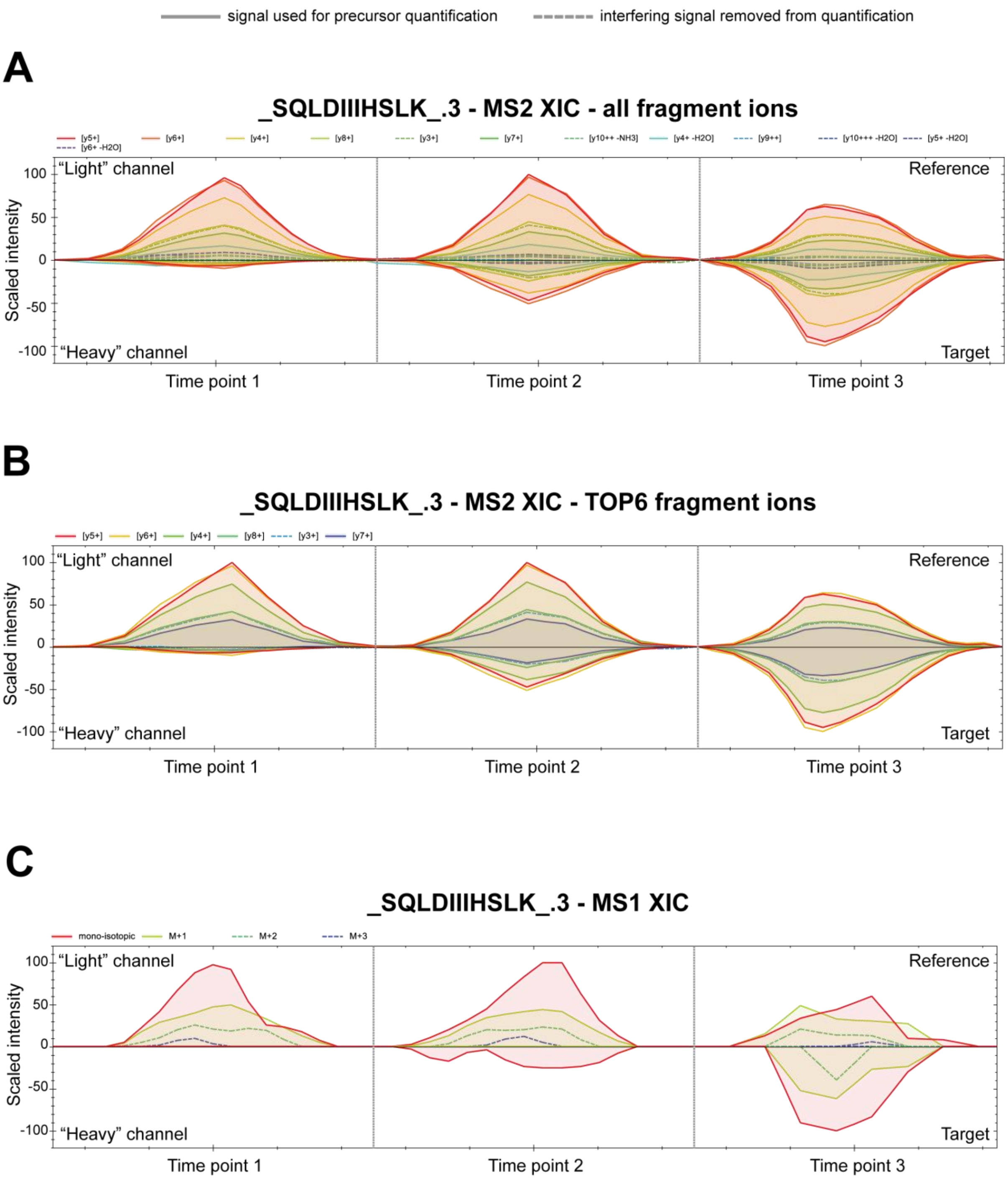
signal used for precursor quantification
interfering signal removed from quantification
A
SQLDIIIHSLK.3 - MS2 XIC - all fragment ions
[y5+]
[y6+]
[y4+]
[y8+]
[y3+]
[y7+]
[y10++ -NH3]
[y4+ -H2O]
[y9++]
[y10+++ -H2O]
[y5+ -H2O]
[y6+ -H2O]
"Light" channel
Reference
"Heavy" channel
Target
Scaled intensity
100
50
0
-50
-100
Time point 1
Time point 2
Time point 3
B
SQLDIIIHSLK.3 - MS2 XIC - TOP6 fragment ions
[y5+]
[y6+]
[y4+]
[y8+]
[y3+]
[y7+]
"Light" channel
Reference
"Heavy" channel
Target
Scaled intensity
100
50
0
-50
-100
Time point 1
Time point 2
Time point 3
C
SQLDIIIHSLK.3 - MS1 XIC
mono-isotopic
M+1
M+2
M+3
"Light" channel
Reference
"Heavy" channel
Target
Scaled intensity
100
50
0
-50
-100
Time point 1
Time point 2
Time point 3

Figure S7

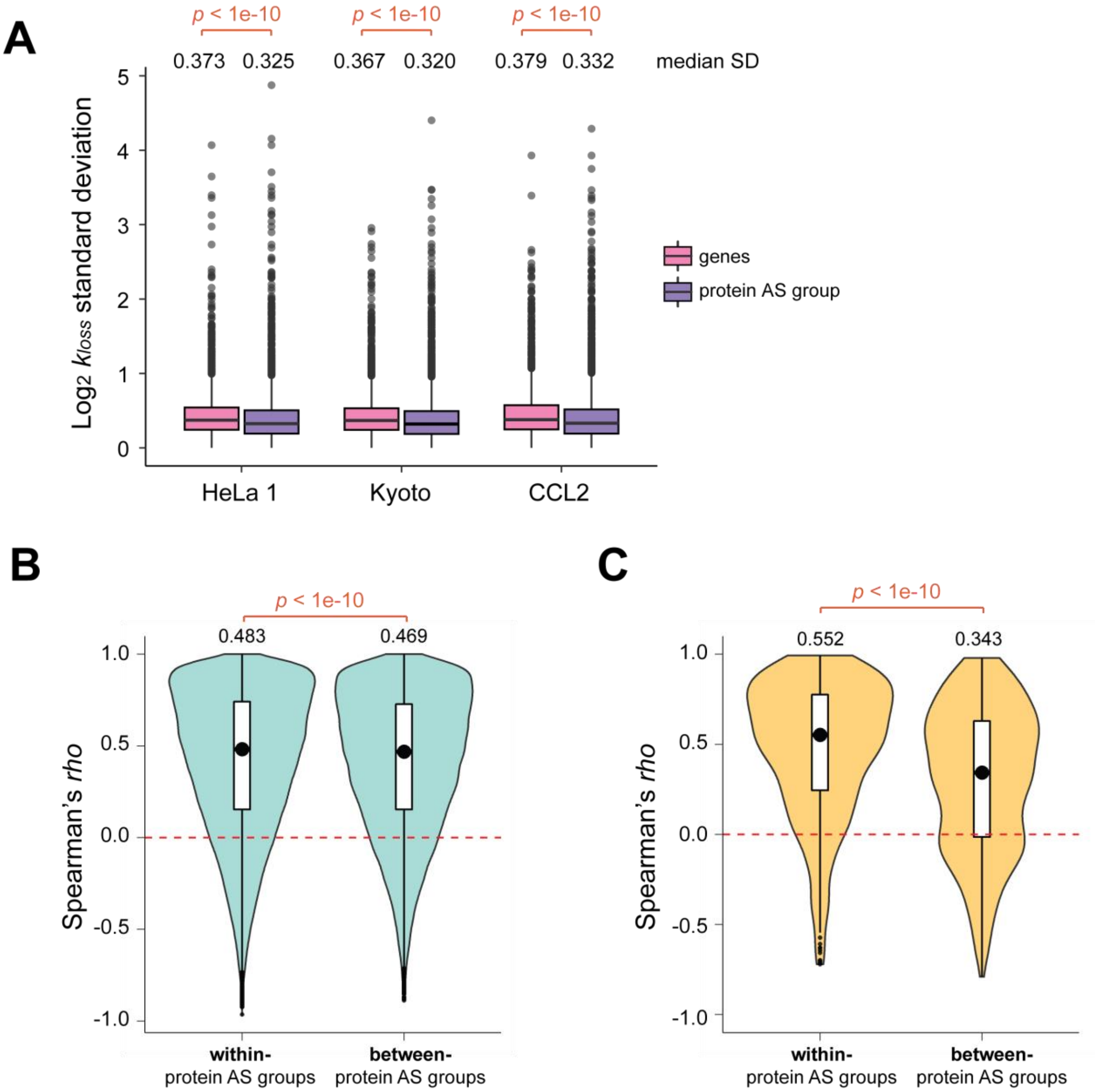
A
p < 1e-10
p < 1e-10
p < 1e-10
0.373 0.325
0.367 0.320
0.379 0.332
median SD
Log2 kloss standard deviation
5
4
3
2
1
0
genes
protein AS group
HeLa 1
Kyoto
CCL2
B
p < 1e-10
0.483
0.469
Spearman's rho
1.0
0.5
0.0
-0.5
-1.0
within-
protein AS groups
between-
protein AS groups
C
p < 1e-10
0.552
0.343
Spearman's rho
1.0
0.5
0.0
-0.5
-1.0
within-
protein AS groups
between-
protein AS groups

Figure S8

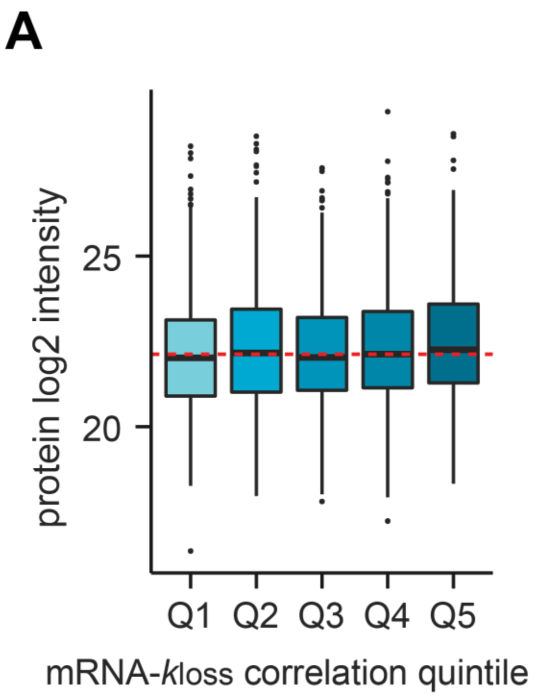
A
B
protein log2 intensity
25
20
Q1 Q2 Q3 Q4 Q5
mRNA-kloss correlation quintile
mRNA log2 FPKM
10
5
0
−5
Q1 Q2 Q3 Q4 Q5
mRNA-kloss correlation quintile
