# Supplementary material for: Isoform‐resolved correlation analysis between mRNA abundance regulation and protein level degradation
Source: Mol Syst Biol. 2020 Mar 16;16(3):e9170. doi: 10.15252/msb.20199170 (PMC7073818; doi:10.15252/msb.20199170)
Supplement: Supplementary file 2 — Expanded View Figures PDF [file MSB-16-e9170-s002.pdf]

# Expanded View Figures

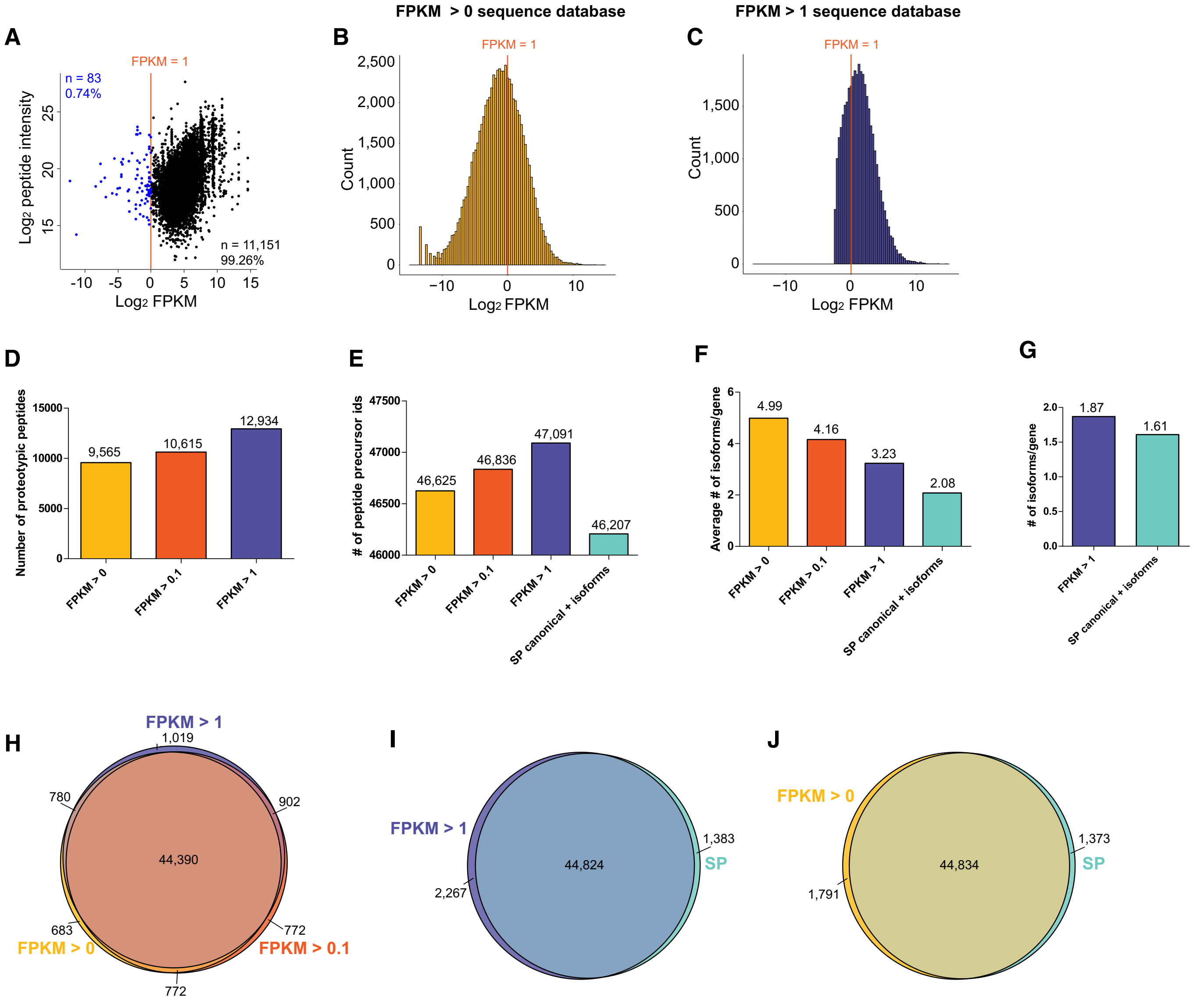


**Figure EV1. Quantitative comparison of results acquired with different protein FASTA DB.**

A Detectability of unique peptides translated from very low-abundant transcripts (blue dots) expressed below a FPKM of 1 (red line). The mRNA data were analyzed using the FPKM > 0 FASTA DB in fourteen HeLa DIA files.

B, C Average $\log_2$ FPKM distribution of transcripts included in the FPKM > 0 (B) and FPKM > 1 (C) databases.

D, E Number of unique (proteotypic) peptides (D) and peptide precursors (E) quantified using FASTA DB with different FPKM cutoffs.

F Average number of alternative splicing isoforms (transcript IDs) per gene ID included in the FASTA DB.

G Average number of alternative splicing isoforms per gene ID quantified using indicated FASTA DB.

H–J Overlap between peptide precursor IDs quantified using indicated FASTA DB.

Data information: (D, E, H–J) Three HeLa 1 pSILAC samples were analyzed using libraries created from five HeLa 1 raw files and different FASTA DB. The Swiss-Prot canonical and isoforms sequence database was downloaded from UniProtKB.


© 2020 The Authors

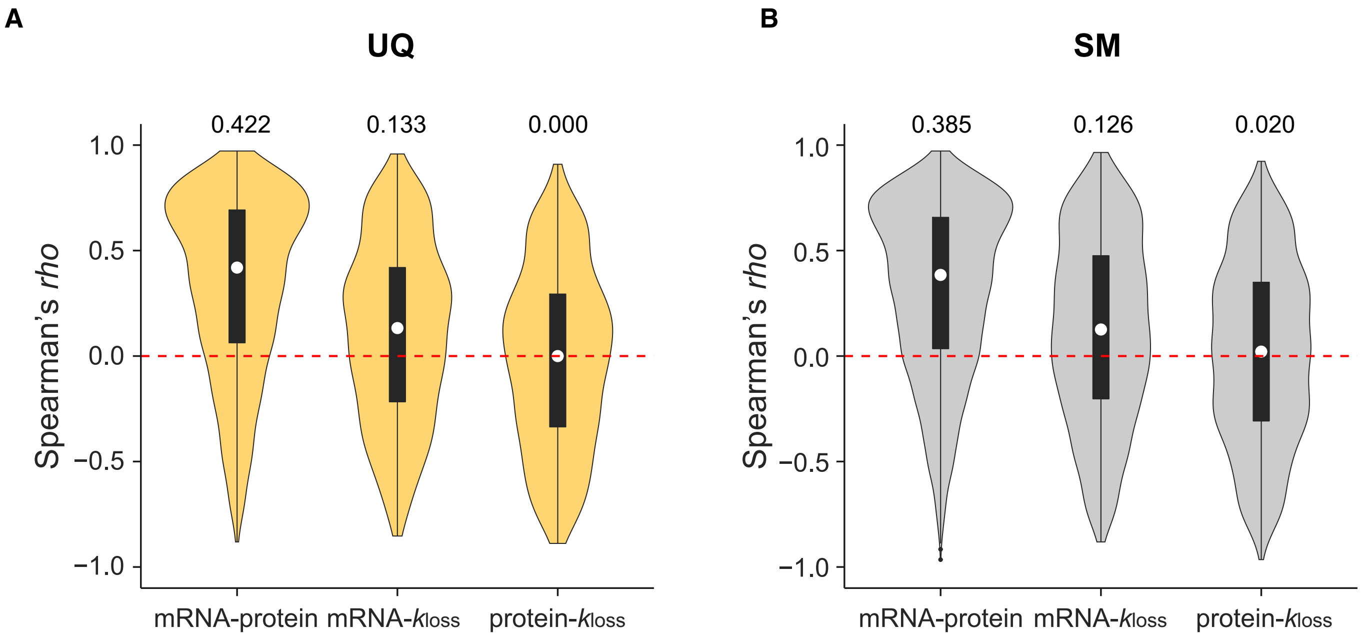


**Figure EV2. Isoform-resolved protein-specific Spearman's correlation analysis using the unique and shared major proteins.**

A, B Distribution of isoform-specific Spearman's ρ of the unique (A) and shared major (B) proteins. The shape of the violin shows the distribution of the data; the white dots and numbers above the plots indicate median values. *n* = 1,739, 885, 885 (left to right) and *n* = 5,645, 2,895, 2,895 (left to right) for UQ and SM, respectively.

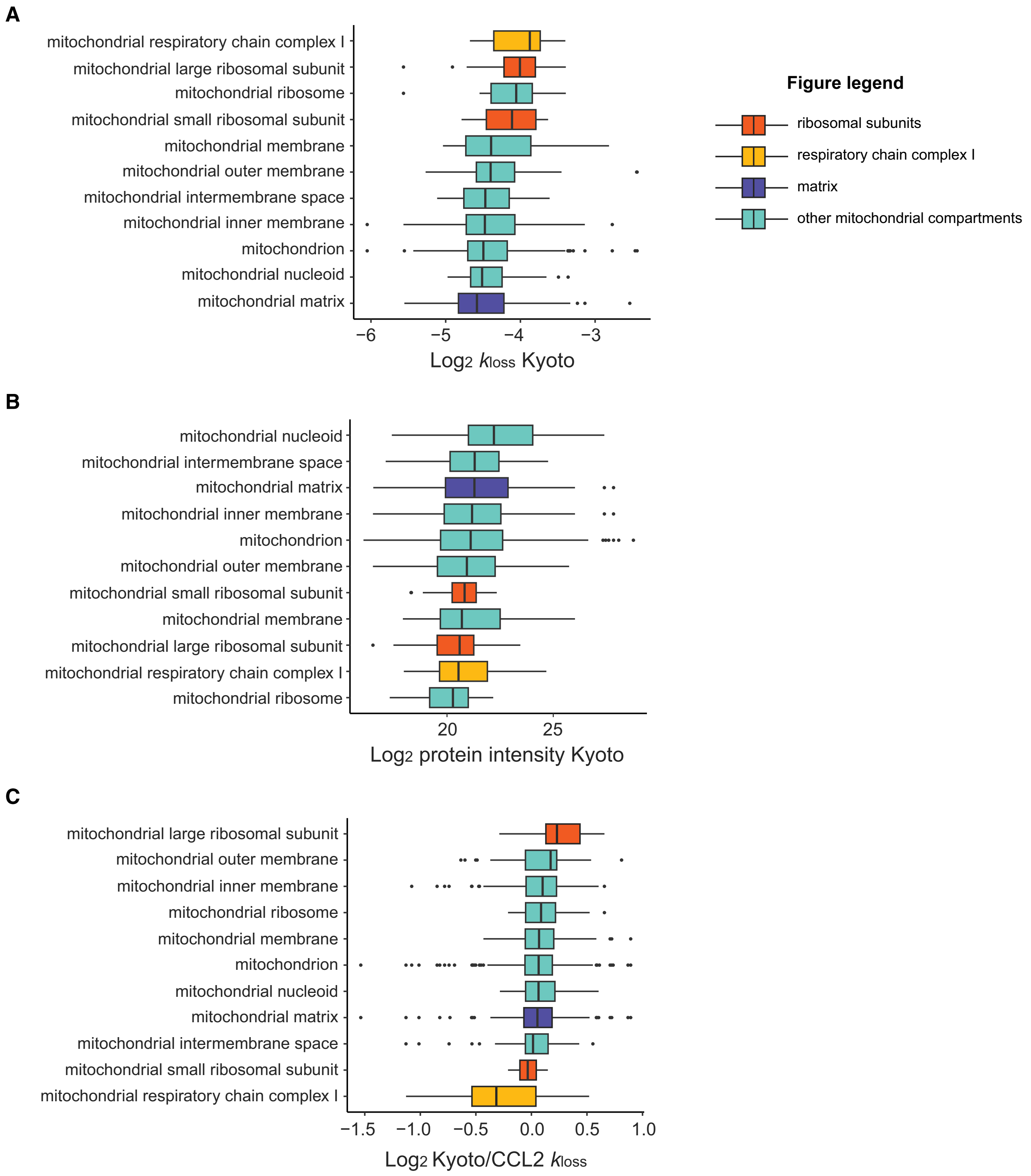


**Figure EV3. The absolute and relative protein degradation rate of mitochondrial compartments.**

A–C Absolute-scale $k_{loss}$ (A), absolute-scale protein intensity (B), and relative-scale $k_{loss}$ (C, i.e., $log_2$ FC Kyoto/CCL2) distributions for proteins included in the selected gene ontology categories (GOCC) related to mitochondria are depicted. Examples discussed in the main text are highlighted with distinctive colors. Box borders represent the $25^{th}$ and $75^{th}$ percentiles, bar within the box represents the median, and whiskers represent the minimum and maximum value within 1.5 times of interquartile range. *n* = 14, 29, 13, 11, 13, 32, 31, 156, 393, 28, and 127, from to bottom as sorted in (A).


© 2020 The Authors

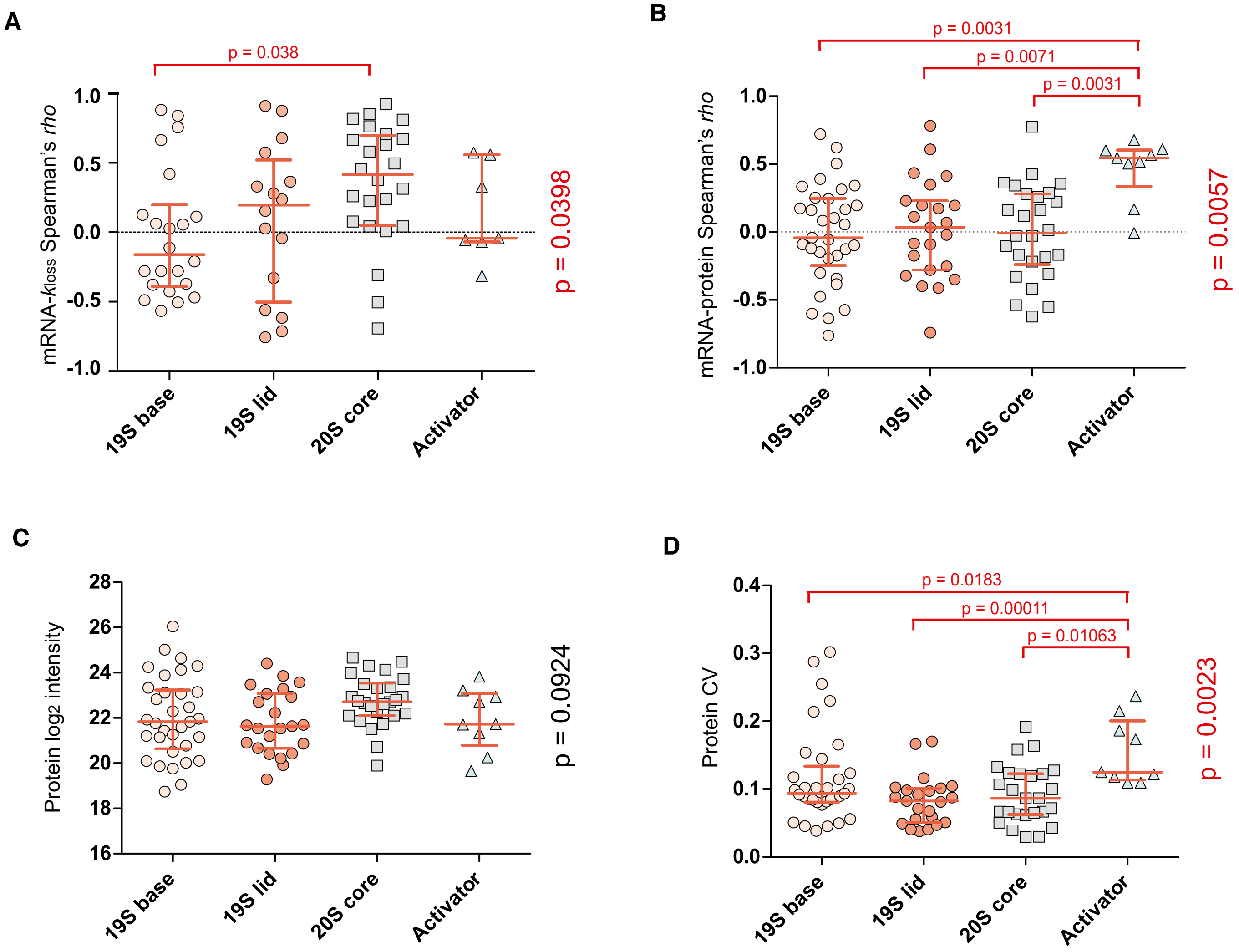


**Figure EV4. Expanded overview of the proteasome subunits (KEGG: Proteasome).**

A–D The 19S regulatory subunit of the 26S proteasome was divided into two groups (base and lid) and the "Activator" includes proteasome activator subunit proteins for further dissection of subunit-specific mRNA–$k_{loss}$ correlation (A), mRNA–protein correlation (B), protein abundance (C), and protein CV (D) within the proteasome complex. Kruskal–Wallis test *P*-values are shown on the right side, pairwise comparisons were performed using pairwise Wilcoxon test with Benjamini–Hochberg correction. The red lines indicate median with interquartile range. The number of protein AS isoform groups is indicated by the number of dots.

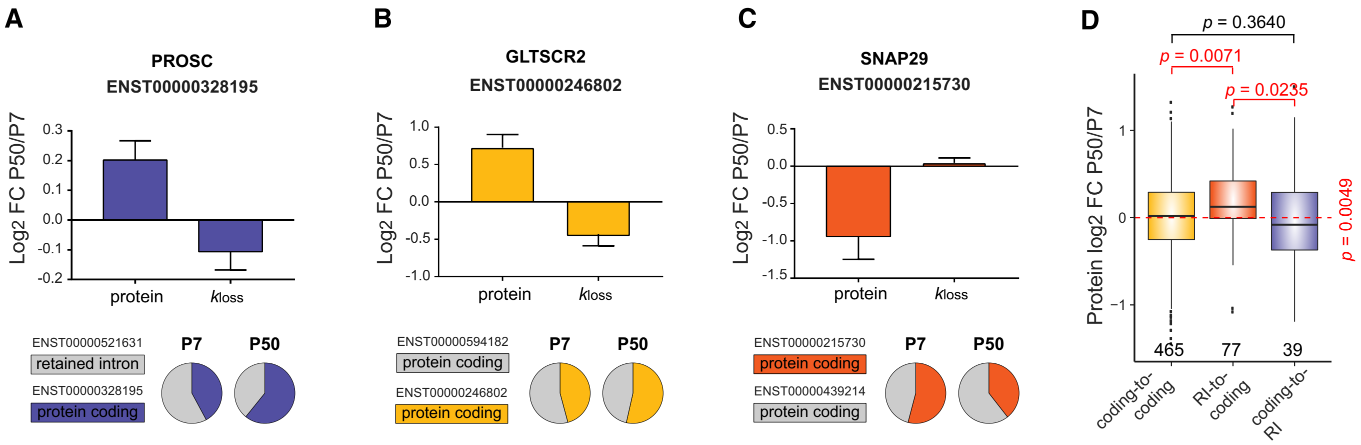


**Figure EV5. Selected examples of alternative splicing switch events translated to protein level.**

A PROSC underwent a switch event from RI to protein coding. The bar charts indicate protein and $k_{loss}$ $log_2$ FC between HeLa CCL2 P50 and P7. The pie charts show a relative expression of the two splicing isoforms in P7 and P50. The protein coding splicing isoform major in P50 is marked by purple color. Number of peptide level values: $n$ = 5 (protein), $n$ = 2 ($k_{loss}$). Bars and error bars correspond to mean ± SEM.

B, C GLTSCR2 (B) and SNAP2 (C) both underwent a switch event from protein coding to protein coding (whereas the major coding AS changed). The bar charts indicate protein and $k_{loss}$ $log_2$ fold change between P50 and P7 HeLa CCL2 cells. The pie charts show relative expression of the two splicing isoforms in P7 and P50. In (A), the protein coding isoform marked by yellow is major in P50, which correlates with P50/P7 $log_2$ fold change $> 0$. In (B), the protein coding isoform marked by red is major in P7, which correlates with P50/P7 $log_2$ fold change $< 0$. Number of peptide level values: $n$ = 4 and 3 (GLTSCR2) and $n$ = 7 and 3 (SNAP29) for protein and $k_{loss}$, respectively. Bars and error bars correspond to mean ± SEM.

D Distribution of P50/P7 protein $log_2$ FC of AS isoforms undergoing a switch event. Switch events "retained intron-to-coding" and "coding-to-retained intron" are compared to the "coding-to-coding" switch events as a control group. The $P$-value (right) was calculated using Kruskal–Wallis test, and the pairwise comparison $P$-values from the Dunn's test. The numbers (bottom) indicate the number of values in each group. RI, retained intron. Box borders represent the $25^{th}$ and $75^{th}$ percentiles, bar within the box represents the median, and whiskers represent the minimum and maximum value within 1.5 times of interquartile range. The dashed red line indicates zero.


© 2020 The Authors
